# Children’s Responses to Peer Conflict Scenarios: Feasibility and Initial Psychometric Properties of an Interactive Virtual Reality Assessment in a Clinical and Non-Clinical Sample

**DOI:** 10.1007/s10802-026-01481-8

**Published:** 2026-07-03

**Authors:** Simon Klos, Celina Eva-Maria Müller, Sophie Burwick, Manfred Döpfner, Anja Görtz-Dorten

**Affiliations:** 1 Center for Child and Adolescent Cognitive Behavior Therapy (CEKIP), Faculty of Medicine and University Hospital Cologne, University of Cologne, Pohligstr. 9, 50969 Cologne, Germany; 2 Department of Child and Adolescent Psychiatry, Psychosomatics and Psychotherapy, Faculty of Medicine and University Hospital Cologne, University of Cologne, Robert-Koch-Str. 10, 50931 Cologne, Germany

**Keywords:** Virtual reality, Children, Assessment, Aggressive behavior, Psychometric properties

## Abstract

Exploring responses to everyday conflict scenarios is important for understanding the development of aggressive behavior in children and for clinical practice. Current research suggests that simulations within virtual reality (VR) improve traditional approaches. We expanded this research by developing a VR-based assessment (VR test) comprising three ambiguous and three hostile peer-related conflict scenarios. This pilot study examined the test’s feasibility and psychometric properties in 8–12-year-old boys, including a clinical sample with aggressive behavior problems (*n* = 42) and a non-clinical sample (*n* = 40). During the VR test, behavioral responses were observed and social information processing (SIP) explored. Additionally, we collected reports of peer-related aggression and disruptive behavior symptoms from parents, children, and teachers. Good feasibility of the test was demonstrated by various indicators (e.g., session duration, appeal, immersion). Internal consistencies of the aggressive behavioral response and SIP scales suggested mostly good reliability of the VR test scales. Intercorrelations between scale scores indicated partial overlaps (*r* = .28–.84). Discriminant validity of the VR test scenarios was demonstrated by higher scale scores for hostile than for ambiguous scenarios and higher scores for ambiguous scenarios compared to a neutral scenario (all *p* < .001). Differences between the clinical and non-clinical sample revealed discriminant validity of VR test scales (*d* = 0.57–1.76). With a few exceptions, correlations of the VR test scales with multi-informant ratings indicated convergent validity (*r* = .02–.71). Overall, these findings suggest feasibility and provide initial evidence of adequate psychometric properties. Further research is needed to ensure psychometric robustness and utility. Study registration: https://www.drks.de/ (Number = DRKS00032850, registration date = 2024/01/30, retrospectively registered).

**Supplementary Information:**

The online version contains supplementary material available at 10.1007/s10802-026-01481-8.

## Introduction

Occasional oppositional, aggressive, and rule-breaking behavior is a normative part of child development. However, such behavior becomes problematic if it occurs frequently, with high intensity, and results in substantial functional impairment (e.g., at home or in school). Oppositional defiant disorder (ODD) and conduct disorder (CD) – characterized by persistent patterns of defiant, aggressive, or rule-breaking behavior (American Psychiatric Association, 2013) – are among the most common mental disorders in children and adolescents, with an estimated worldwide prevalence of 5.7% (Polanczyk et al., 2015). In view of various negative long-term outcomes of conduct problems, including poor educational and occupational outcomes, general health problems, and criminal behavior (Bevilacqua et al., 2018), it is crucial to understand the etiology of ODD and CD, improve assessment procedures, and intervene at an early stage.

Research has demonstrated that dysfunctional social information processing (SIP) is an important risk factor for the development and maintenance of aggressive behavior in children (De Castro & van Dijk, 2018). Therefore, it is essential to develop and evaluate assessment procedures that provide information about SIP. An understanding of the influence of SIP on disruptive behavior can help reveal the child’s own perspective, prevent recurrence, and improve treatment (De Castro & van Dijk, 2018). Moreover, an assessment of SIP may inform the development of tailored interventions, support treatment monitoring, and might be a valuable outcome measure for treatment effectiveness studies. Indeed, such an assessment is particularly relevant given that psychotherapeutic interventions addressing SIP (e.g., Lochman & Wells, 2004) are widely used in practice, and research has demonstrated the effectiveness of school-based prevention programs focusing on SIP (e.g., Dodge & Godwin, 2013).

SIP in children is typically assessed using hypothetical peer-related social scenarios to address and measure peer-related aggression, which are presented to children via pictures (Niestroj et al., 2023), audio recordings (De Castro et al., 2005), or videos (Kupersmidt et al., 2011). Children are asked to imagine that the hypothetical scenarios would happen to them in real life and are then asked questions that assess SIP. These questions mostly rely on theoretical models. According to one established model, developed by Crick and Dodge (1994), a behavioral response to a social situation (*Behavioral Enactment)* is a product of a sequence of SIP steps that refer to how information is encoded (*Encoding of Cues*), how it is interpreted (*Interpretation of Cues*), which goals are selected (*Clarification of Goals*), what responses are generated (*Response Access or Construction*), and how these responses are evaluated (*Response Decision*). Among these steps, the Interpretation of Cues, which includes *Hostile Attribution Bias*, has been well studied, with a meta-analysis finding a small to moderate relation between aggressive behavior and hostile attribution (Verhoef et al., 2019). Since this bias refers to the tendency to interpret ambiguous social cues as intentionally hostile, assessment procedures commonly use so-called *ambiguous scenarios*, in which the intention of a perpetrator is unclear.

### Limitations of Traditional Assessments

However, traditional SIP assessment procedures have several limitations. First, pictures, audio recordings, or videos, as typically used in SIP assessment, do not induce the feeling of being present and involved in scenarios (immersive experience) and may therefore be limited in the degree to which they trigger children’s emotional engagement. Accordingly, the arising emotions may differ significantly from the emotions that children would likely feel in real-life situations (Li et al., 2013). As a consequence, it is difficult to transfer the results of such procedures to everyday life, and ecological validity is limited (Verhoef et al., 2022). Second, when using these traditional approaches, behavioral responses to conflict scenarios can only be asked about hypothetically, and researchers and clinicians do not generally conduct observational ratings of children’s behavioral responses. Therefore, the final step of the aforementioned SIP model (Behavioral Enactment) cannot be analyzed based on observed behavior. In view of findings that social competence training benefits from employing realistic role-play scenarios (Goertz-Dorten et al., 2023), an adequate assessment of behavioral responses may help problematic behaviors to be directly addressed within treatment sessions, thus also potentially fostering the individualization of treatment. Third, the exclusive use of ambiguous scenarios results in a lack of information about cognitions, emotions, and behavioral responses in scenarios that are clearly hostile. Large-scale surveys in school settings reveal that incidents of peer-related aggression are very common (Healy et al., 2020). This peer-related aggression involves both reactive aggression and proactive provocations and bullying. Assessment procedures that do not include clearly hostile scenarios thus fail to capture information about children’s everyday life. Therefore, to achieve a more comprehensive assessment of response evaluation skills, researchers have called for the inclusion of hostile scenarios in assessment tools (Kupersmidt et al., 2011; Li et al., 2013). Indeed, some studies have already implemented such scenarios, for example in bullying research (Smalley & Banerjee, 2014) or forensic studies (Westerveld et al., 2025).

### Potential of Virtual Reality (VR)

The use of VR represents a promising approach to address the above-mentioned limitations. First, research on VR-based assessments of aggressive SIP in children has shown that VR-based scenarios elicit greater emotional engagement and immersion experience compared to audiotaped scenarios, leading to increased predictive validity of real-life aggression and motives for aggression (Verhoef et al., 2022). Second, according to Lobbestael and Cima (2021), VR offers several advantages for assessing behavioral responses in conflict scenarios. For example, the immersive experience in VR may lead to a greater sense of privacy compared to a typical laboratory environment, as the experimenter’s presence is not directly perceived and participants' responses are therefore more likely to mirror those in everyday life situations. Additionally, the use of VR can be highly standardized, while simultaneously being highly modifiable, enabling an assessment that is experimentally controlled but also flexible. Moreover, as VR does not involve any real harmful behavior towards humans (e.g., physical aggression), ethical concerns are reduced. Previous studies in children have demonstrated that VR is both feasible and ethically acceptable for the assessment of aggressive behavior (Verhoef et al., 2022) and for the modification of aggressive behavior through role-play interventions (Alsem et al., 2023). Nevertheless, while immersive simulations of conflict scenarios provide the advantage of inducing emotional engagement, thereby helping to capture aggressive responses that closely resemble real-life behavior, this emotional engagement can also lead to discomfort, particularly in vulnerable groups (e.g., clinical populations, younger participants). Therefore, efforts to achieve ecological realism must be carefully balanced with the responsibility to protect participants’ well-being, and appropriate safeguards should be in place (Hernandez-Pena et al., 2026). Third, studies in adolescents and adults provide evidence that clearly hostile VR-based scenarios improve the prediction of aggressive behavior (Terbeck et al., 2022; Westerveld et al., 2025). Since VR provides the opportunity to simulate challenging situations that are difficult to reproduce in real life, while eliciting realistic behavior despite participants’ awareness of the virtual setting (Freeman et al., 2017), VR can be seen as a promising tool for assessing cognitions, emotions, and behaviors in hostile scenarios in children.

### Current Study

A recent review providing a comprehensive overview of VR-based studies in aggression research highlighted the added value of VR as a context-sensitive framework that enables aggressive behavior to be captured in real time within ecological settings, and emphasized the need for research in children using experimental, task-oriented approaches (Hernandez-Pena et al., 2026). In children, VR-based peer-related conflict scenarios have so far been used to examine aggressive behavioral responses and SIP in reactive and proactive aggression, with promising results, especially for the assessment of reactive aggression in ambiguous conflict scenarios (Verhoef et al., 2022). To the best of our knowledge, only one study in children has systematically varied the ambiguity in conflict scenarios by contrasting ambiguous and clearly hostile scenarios in order to measure reactive aggressive behavior and dysfunctional SIP (Hummer et al., 2023). However, this study was limited in scope, as it focused solely on assessing the hostile attribution bias while neglecting several key SIP components (e.g., response access or evaluation). Moreover, it did not assess aggressive behavioral responses during the interaction, and it relied on a single scenario per condition. To extend previous research, the present study aimed to provide an initial evaluation of an interactive VR-based assessment for children that enables the observation of aggressive behavioral responses while simultaneously capturing multiple dysfunctional SIP dimensions in various peer conflict scenarios, and explicitly differentiates between ambiguous and clearly hostile scenarios. Therefore, we developed a VR introductory session, which included a neutral scenario without conflict potential, and the VR-based assessment (VR test), which consisted of three ambiguous and three clearly hostile scenarios. Both sessions were conducted with boys from a clinical sample with aggressive behavior problems and boys from a non-clinical sample.

In the first step, we aimed to explore the feasibility of the VR test, with no specific hypotheses formulated a priori. The concept of feasibility was applied in a broader sense, as we explored not only traditional feasibility indicators (e.g., session duration) but also participants’ experiences of and responses to the VR test and its scenarios, which we viewed as essential for a comprehensive evaluation of the assessment. Specifically, we analyzed session feasibility, participants’ perceived appeal of the VR test, and their immersive experience of the test. We further evaluated whether the scenarios and reactions to them were perceived as realistic, emotionally engaging, and capable of eliciting aggressive behavioral responses.

In the second step, we aimed to gather preliminary evidence regarding the psychometric properties of the VR test. We explored internal consistencies and intercorrelations of the VR test scales (aggressive behavioral response scales, SIP scales). Next, we examined two aspects of discriminant validity. We investigated the extent to which the VR test scale scores differed between neutral, ambiguous, and hostile scenarios, hypothesizing, a priori, higher scale scores in hostile scenarios than in ambiguous scenarios as well as higher scale scores in ambiguous scenarios compared to a neutral scenario. Moreover, we examined differences in the VR test scale scores between the clinical and non-clinical sample, hypothesizing, a priori, higher scores in the clinical sample. Finally, we explored convergent validity by analyzing correlations between the VR test scales and multi-informant ratings of clinical symptoms and peer-related aggression observed in daily life.

## Methods

### Sample

Data were collected from a clinical sample of boys with a confirmed diagnosis of ODD or CD (*n* = 42) and a non-clinical sample of boys (*n* = 40). Participants were aged 8–12 years at the first study visit (*M* = 10.46, *SD* = 1.39). Recruitment took place between August 2023 and February 2025. The clinical sample was recruited from the outpatient unit of the Center for Child and Adolescent Cognitive Behavior Therapy (CEKIP) at the University Hospital Cologne, Germany. A subsample (*n* = 32) was recruited to take part in VR-based social skills training. As the study evaluating the social skills training was limited to a predefined number of participants, for the present study, we expanded our clinical sample by including children who were receiving standard treatment at the outpatient unit (*n* = 10). All boys in the clinical group had been diagnosed with hyperkinetic conduct disorder (ICD-10 code: F90.1), conduct disorder (ICD-10 code: F91), or mixed disorders of conduct and emotions (ICD-10 code: F92). The non-clinical sample was recruited through schools, youth organizations, and the outpatient unit (e.g., children of employees and their friends). For inclusion in the non-clinical group, boys needed to show no indication of a disruptive behavior disorder or peer-related aggression (stanine score < 7), as determined by parent ratings on the *Symptom Checklist for Disruptive Behavior Disorders* (SCL-DBD; Doepfner & Goertz-Dorten, 2017) and the *Questionnaire for Aggressive Behavior of Children* (FAVK; Goertz-Dorten & Doepfner, 2021). Following a multi-informant approach, ratings were obtained from 82 parents, 82 children, and 64 teachers, with each parent report corresponding to one child report. Fewer teacher reports were available because participation required both family consent and teacher agreement. The teacher data were clustered, as some teachers rated multiple children (*n* = 11 teacher ratings with cluster sizes: 2 [*n* = 8], 3 [*n* = 2], 4 [*n* = 1]). Two boys were excluded due to cybersickness symptoms during the VR introductory session, assessed using four modified items (nausea, disorientation, oculomotor problems, general discomfort) from the *Simulator Sickness Questionnaire* (Kennedy et al., 1993). No children in either the clinical or non-clinical sample were excluded due to low intelligence. A detailed sample description is provided in the supplementary materials (see Table S1).

### Procedure

Parents of participants from the clinical sample underwent a telephone screening to check the inclusion and exclusion criteria (e.g., presence of aggressive behavioral problems, no evidence of epilepsy). Subsequently, disruptive symptoms of children were assessed using the diagnostic interview *ILF-EXTERNAL* (Goertz-Dorten et al., 2022b) conducted with the parents. In parallel, children completed the *Culture Fair Intelligence Test* (CFT; Weiß, 2006, 2012) to check the exclusion criterion of low intelligence (IQ < 80). For children already receiving treatment at the outpatient unit, results from existing intelligence assessments were used. Parents of participants from the non-clinical sample likewise underwent a telephone screening to check the inclusion and exclusion criteria (e.g., absence of aggressive behavioral problems, no evidence of epilepsy). In the non-clinical sample, the exclusion criterion of a low IQ was evaluated based on a clinical rating which included anamnestic information from the telephone screening, school reports, and the clinical impression during the first study visit. Moreover, for both samples, multi-informant ratings (parents, children, teachers) were gathered via questionnaires (e.g., FAVK, SCL-DBD). All eligible children from both samples received a VR introductory session, during which the exclusion criterion of cybersickness symptoms was assessed. At the following appointment, participants completed the VR test. Boys from the non-clinical sample received a €15 voucher as compensation. The study was approved by the Ethics Commission of Cologne University’s Faculty of Medicine. Prior to participation, written informed consent was obtained from all participating parents and teachers, and written and verbal informed assent was obtained from all participating children.

### Interactive VR

#### Material

The VR sessions were conducted using VR goggles (Oculus Rift S, resolution: 1280 × 1440 per eye, diagonal field of view: 101.95°, refresh rate: 80 Hz) and two VR controllers (Oculus Touch). We used the interactive VR software Social Worlds VR-CBT (version 4.1) developed by the company CleVR B.V., which has already provided software for other research projects with similar target groups (Alsem et al., 2021; Verhoef et al., 2021). The software offers the possibility to carry out role-plays between children and virtual avatars. An experimenter controls the gestures and facial expressions of the virtual avatars and talks to the children via a computer-distorted child-like voice. The avatars can respond immediately when children have spoken or made physical contact, ensuring naturalistic interaction. Eight locations that are common in children’s everyday life were used in the scenarios (schoolyard, park, playground, city, bus stop, on the bus, supermarket, basketball court). The scenarios used eleven male, child-like avatars, which varied in clothing, facial features, skin color, and body height (135–150 cm). The participant’s height was standardized to 145 cm. The VR sessions took place in a specially prepared room, in which the children had approximately nine square meters of space to move around freely. Besides this VR walking area, the room also contained a VR control cabin, from which the operator controlled the VR environment, could hear what the child was saying through headphones, and observed the child`s behavior through a window. In addition, there was a workstation with a computer for interviewing the children, and three cameras capturing participants’ behavior from different angles. A schematic visualization of the room is included in the supplementary materials (see Figure S1).

#### Development of VR Scenarios

With the objective of designing age-typical conflict scenarios, the development of the VR test scenarios was primarily informed by two sources: First, we used the *Computer-Based Social Skills Training ScouT* (Goertz-Dorten & Doepfner, 2016), a cognitive-behavioral intervention for children using five peer-related conflict scenarios, which has been shown to be more effective than a supportive resource activation treatment (Goertz-Dorten et al., 2022a). Based on these five scenarios, we developed five scenarios for the VR test: *Untrue Allegation, Social Exclusion, Depreciation, Insult,* and *Physical Threat*. Second, the scenario *Defeat in a Competition* was developed based on insights from the *Taxonomy of Problematic Social Situations* (TOPS; Matthys et al., 2001). Following discussions within the study team, two expert meetings, and trial runs with 21 children, the content of the six conflict scenarios for the VR test was set as follows: In the three ambiguous scenarios, the intentions of the VR avatars could be interpreted either as accidental or as hostile (Defeat in a Competition, Untrue Allegation, Social Exclusion), and in the three hostile scenarios, the avatars acted clearly provocatively (Depreciation, Insult, Physical Threat). The VR introductory session included a neutral scenario (Small Talk in a Supermarket), which contained no conflict potential and was performed for the purpose of practice (e.g., familiarization with the VR environment) and validation (e.g., examining discriminant validity). Descriptions of all scenarios can be found in the supplementary materials (see Figure S2).

#### Implementation of VR Sessions

The VR introductory session was conducted by one study staff member, while the VR test was administered by two study staff members. Three different study staff members served as operators of the VR software (conducting 20, 14, and 8 sessions, respectively, in the clinical sample, and 22, 9, and 9 sessions, respectively, in the non-clinical sample). A storyline was created across both VR sessions. In the VR introductory session, Till Taff, a virtual avatar of the same age as the participant, introduced himself and guided the participant through the virtual world Tiviwo (Till’s Virtual World). The children were familiarized with the different locations that would later be used in the VR test and met other virtual avatars living in Tiviwo, each given a name to facilitate recognition during the VR test. At the end of the session, the neutral scenario was carried out. The VR test session took place approximately one week later and began with a brief introduction by Till Taff, followed by six scenarios presented in a fixed order: three ambiguous scenarios (1. Defeat in a Competition, 2. Untrue Allegation, 3. Social Exclusion) and three hostile scenarios (4. Depreciation, 5. Insult, 6. Physical Threat). During the VR test session, the children were instructed to imagine that the scenarios would happen to them in real life. If avatars were already familiar from the VR introductory session, children were asked to imagine them as familiar peers (e.g., classmates); otherwise, they were to imagine them as unfamiliar children (see Figure S2 for further details on avatar familiarity per scenario). The first, third, and fourth scenario of the VR test involved two avatars, while the remaining scenarios included one avatar. The third and fourth scenarios were conducted in a seated position, while the remaining scenarios were performed standing. Each scenario followed the same script, ensuring a high degree of standardization of the VR test: (1) introduction to the upcoming scenario in a low-stimulus waiting room, (2) VR interaction in which avatars performed two standardized actions (verbal or/and gesture) requiring two behavioral responses, and (3) immediate post-scenario interview assessing emotional engagement and SIP scales (*Cue Interpretations*, *Goals*). After all scenarios had been completed in VR, participants were asked further questions to assess the immersion experience, appeal, ecological validity, and SIP scales (*Response Access*, *Response Evaluations*). The questions were split into two parts because trial runs revealed that repeatedly removing and replacing the VR headset after each scenario disrupted the immersion experience and was perceived as annoying. Moreover, only a limited number of questions could feasibly be answered while the children were wearing the VR headset. Regarding the interaction within VR during the conflict scenarios, we chose to use two standardized actions of the avatars as an escalation of the conflict, requiring two behavioral responses per participant, since our trial runs showed that using only one action resulted in very brief interactions, which did not resemble real-life conflicts, and implementing more than two actions was difficult due to the standardization of scenario implementation and ethical considerations. Figure S3 illustrates the sequence of the VR test scenario Depreciation (see supplementary material).

In accordance with ethical considerations, multiple safeguards were implemented to minimize short- and long-term risks to participants, including the following elements: Informed consent procedures ensured that both children and their parents fully understood the study and had the opportunity to ask questions. An introductory session was conducted to familiarize participants with the procedure. The VR environment (“Tiviwo”) was presented as a fictional, child-appropriate setting in which conflict scenarios could be safely experienced. The conflict scenarios were developed using very brief interaction sequences. Throughout the VR test, children were permitted to take breaks and to discontinue participation at any time. Continuous monitoring by study members was maintained during all sessions. All scenarios were systematically debriefed with the children, and difficult scenarios were discussed. Both participants and their parents were invited to report any concerns following the sessions.

### Measure

#### Feasibility of the VR Test

Session feasibility was assessed by the study staff members conducting the session. The study staff recorded whether a technical issue occurred, its nature (e.g., battery replacement needed), and the resulting delay in minutes. They also rated the impact of such incidents on a 4-point Likert-type scale ranging from 0 (*no impact on the session*) to 3 (*great impact on the session*). Additionally, the session duration in minutes was noted and the number of completed scenarios was documented.

Appeal was measured using two items at the end of the VR test session (“I liked what we did today,” “I am / would be looking forward to the next session”), which were adapted from Alsem et al. (2021). The wording of the second item depended on whether or not further VR-based social skills training sessions were planned. The items were rated on a 4-point Likert-type scale ranging from 0 (*not at all*) to 3 (*very much*), and an average score was calculated across the two items (*r* = .36).

Immersion experience was measured at the end of the VR test session using five items. Four of these items were adapted from Verhoef et al. (2022) based on previous work by Schubert et al. (1999): “I was totally caught up by the scenarios,” “I had the feeling that the scenarios in VR were happening to me,” “During the VR, it felt like I was actually experiencing the scenarios,” “The events in Tiviwo seemed almost real”*.* The fifth item (“During the scenarios, I had a sense of being in Tiviwo*”*) showed high factor loadings in previous studies (Schubert et al., 2001). The items were rated on a 5-point Likert-type scale ranging from 0 (*strongly disagree*) to 4 (*strongly agree*), and an average score was calculated across the five items (ɑ = .88 [.83, .92]).

Ecological validity was assessed using two items for each VR test scenario (“Do you think something like that could happen to you or another child in real life?,” “Did you behave in this scenario the same way you would with other children your age?”), rated on a 4-point Likert-type scale ranging from 0 (*not at all*) to 3 (*very much*).

Emotional engagement was measured using two items adapted from Verhoef et al. (2022) for each VR test scenario (“How angry did you feel when this happened to you?,” “How much did you care when this happened to you?”), rated on a 4-point Likert-type scale ranging from 0 (*not at all*) to 3 (*very much*) and averaged per scenario. The correlation between the two items varied depending on the scenario (*r* = .49–.68).

#### Outcomes of the VR Test

Aggressive behavioral responses were assessed using observer ratings based on video recordings from three camera perspectives as well as written transcripts of participants’ responses. Each participant displayed two behavioral responses per scenario, which were coded on the following 4-point behaviorally anchored rating scale: 0 = *No aggressive behavior*: no evidence of verbal or physical aggressive behavior; 1 = *Minor aggressive behavior*: provocative or impolite behavior initiating or encouraging a conflict; 2 = *Moderate aggressive behavior*: stronger insults (e.g., swear words) or physically provocative behavior; 3 = *Severe aggressive behavior*: physical threats or acts of physical aggression (e.g., hitting, kicking). An example of the procedure as well as examples of participants’ responses are provided in Figures S3-S4 of the supplementary material. The rating scale was developed based on two expert meetings, trial runs, and discussions in the study team. In addition to the first author, two trained psychotherapists, who were independent of the study team and blinded to group membership, rated all videos. These two additional raters took part in rater training. All raters had at least three years of clinical expertise in the treatment of disruptive behavior disorders. Interrater reliability was calculated using two intraclass correlation coefficients (ICC) following recommendations from Koo and Li (2016). We chose *two-way random-effects models with multiple raters/measurements*, as all subjects were rated by the same set of raters and given that we planned to generalize our results to any raters possessing the same characteristics as the selected raters and to use the mean value of the ratings. Values were calculated for *absolute agreement* and *consistency*, as results from both approaches provide meaningful insights. ICCs ranged from *r* = .74 to *r* = .99 for absolute agreement and from *r* = .74 to *r* = .99 for consistency across behavioral responses and scenarios (see supplementary material Table S2). Ratings were averaged across the raters and behavioral responses for each scenario. Across the different scenarios, the correlation of the average rating for the three raters between behavioral response 1 and behavioral response 2 ranged from *r* = .47 to* r* = 62.

SIP was assessed based on the SIP model by Crick and Dodge (1994). Items were adapted from the *Social Information Processing Application* (SIP-AP) developed by Kupersmidt et al. (2011), translated into German by our study group and used for research purposes. The SIP scales have demonstrated good psychometric properties in community and high-risk samples (Bookhout et al., 2021; Kupersmidt et al., 2011), including adequate internal consistencies (α = .72–.95) and evidence of convergent validity through associations with ratings of aggression, social competence, and rule-breaking behavior obtained from multiple informants. In the present study, four SIP dimensions were assessed for each scenario: *Cue Interpretations* (four items), *Goals* (two items), *Response Access* (three items), and *Response Evaluations* (three items). Items were rated on a 4-point Likert-type scale ranging from 0 (*not at all*) to 3 (*very much*); these scales were treated as exploratory given that the factor structure could not be established. The original items can be found in Kupersmidt et al. (2011). Item characteristics and a description of modifications of the items used in this study can be found in the supplementary materials (see Table S3).

#### Multi-Informant Rating Scales

Peer-related aggression was measured using 25 items from the FAVK rated by parents, teachers, and children (Goertz-Dorten & Doepfner, 2021) on a 4-point Likert-type scale ranging from 0 (*not at all*) to 3 (*very much*). Following the test manual, we formed a *Peer-Related Aggression Total* scale and four subscales: *Disturbance of Social Cognitive Information Processing* (eight items), *Disturbance of Impulse Control* (five items), *Disturbance of Social Skills* (six items), and *Disturbance of Social Interaction* (six items). The first three subscales assess children’s cognitions, emotions and behavior, while the fourth captures social environmental reactions (e.g., by parents) to peer-related aggression. The FAVK has been used as a primary outcome in randomized controlled trials evaluating interventions for children with ODD/CD (e.g., Goertz-Dorten et al., 2022a) and in studies of SIP measures (Niestroj et al., 2023). Scale characteristics for the present study across informants are provided in the supplementary materials (see Tables S4-S6).

ODD/CD symptoms were measured using the SCL-DBD, completed by parents, teachers, and children. The SCL-DBD is part of the *Diagnostic System of Mental Disorders in Children and Adolescents based on the ICD-10 and DSM-5* (DISYPS-III, Doepfner & Goertz-Dorten, 2017). Items were rated on a 4-point Likert-type scale ranging from 0 (*not at all*) to 3 (*very much*). The symptom checklists have demonstrated mostly acceptable psychometric properties in clinical and community samples (Goertz-Dorten et al., 2014; Ise et al., 2014; Klos et al., 2024), including mostly adequate internal consistencies (α = .67–.93) and evidence of discriminant validity by comparing scale scores of children with and without an ODD/CD diagnosis. Following the test manual, we formed an *ODD Total* scale consisting of all eight assessed ODD items. Moreover, consistent with previous research on the factor structure of ODD symptoms (Klos et al., 2024; Rowe et al., 2010), we examined two ODD subscales (*ODD Irritable* scale [three items], *ODD Headstrong* scale [five items]). Additionally, we used an abbreviated *CD* scale consisting of five symptoms (“Physical fights with other children,” “Bullies, threatens, or intimidates,” “Cruel to animals,” “Lies,” “Steals without confrontation”). We decided to use this abbreviated scale since research in a similar sample with externalizing behavior problems and the same age structure as in the present study used the same item set for this questionnaire (Thoene et al., 2021), and the abbreviated scale excludes symptoms indicating more serious conduct problems (e.g., “Uses weapons in fights,” “Fire setting*”*). In line with the test manual, one item (“Cruel to animals*”*) was omitted from teacher ratings, as teachers cannot reliably evaluate this behavior, resulting in a 4-item teacher-report *CD* scale. Scale characteristics for this study across informants are reported in the supplementary materials (see Tables S4-S6).

### Data Analysis Plan

All statistical analyses were conducted using IBM SPSS Statistics (version 29). Given the low proportion of missing data across the different measures, no imputation procedures were applied. Analyses were performed on all available data per analysis, which resulted in slightly varying sample sizes depending on variable completeness. For each analysis, the number of valid cases is provided. In line with previous research (Goertz-Dorten et al., 2022a), all scale scores were computed for participants with less than 10% missing item values on the respective scale.

In a first step, we examined the feasibility of the VR test using descriptive analyses (means, standard deviations) for session feasibility indicators, appeal, immersion experience, ecological validity, and emotional engagement in the VR test scenarios, and conducted exploratory comparisons of immersion experience and emotional engagement between the clinical and non-clinical sample using Welch’s t-tests. To evaluate the elicitation of aggressive behavioral responses, frequencies were reported for each scenario. Mean ratings across raters were categorized (< 0.50 = *No aggressive behavior*; ≥ 0.50 and < 1.50 = *Minor aggressive behavior*; ≥ 1.50 and < 2.50 = *Moderate aggressive behavior*; ≥ 2.50 = *Severe aggressive behavior*). To explore inter-operator effects, several Kruskal–Wallis tests were conducted separately for the clinical and the non-clinical sample, with operator as the between-subject factor and the VR test scales as dependent variables.

In a second step, we sought to gain initial insights regarding the psychometric properties of the VR test. We began by evaluating the scale characteristics of the aggressive behavioral response and SIP scales across ambiguous and hostile scenarios. Internal consistencies were estimated using Cronbach’s α (Cronbach, 1951), with values ≥ .70 considered as acceptable (Nunnally & Bernstein, 1994); McDonald’s ω (McDonald, 1999) is provided in the supplementary material. Additionally, we investigated corrected item-total correlations, considering values *r*_it_ > .30 as acceptable (Field, 2018), and explored intercorrelations among VR test scales. Next, we examined two aspects of discriminant validity: First, we examined the extent to which VR test scale scores differed between neutral, ambiguous, and hostile scenarios in order to explore the discriminant validity of the VR test scenarios. For this purpose, we conducted several one-way repeated measures analyses of variance (rm-ANOVA) with scenario type (neutral, ambiguous, hostile) as the within-subject factor and the VR test scales as dependent variables. Sphericity was evaluated using Mauchly’s test (*p* < .05), and Greenhouse–Geisser correction was applied when the assumption of sphericity was violated. We calculated Cohen’s *f*, with effect sizes interpreted as small (*f* = 0.10), medium (*f* = 0.25 medium), or large (*f* = 0.40) in line with Cohen (2013). Bonferroni post hoc tests were applied. Second, we examined the discriminant validity of the VR test scales by comparing scores between the clinical and non-clinical sample using Welch’s t-tests as the standard option (Rasch et al., 2011). Effect sizes were calculated using Cohen’s* d*, and interpreted as small (*d* = 0.20), medium (*d* = 0.50), or large (*d* = 0.80) in line with Cohen (2013). Lastly, convergent validity was examined using Pearson product-moment correlations of the VR test scales with the peer-related aggression scales and the ODD/CD symptom scales rated by parents, teachers, and children, with p-values adjusted using the Benjamini–Hochberg procedure to control the false discovery rate (Benjamini & Hochberg, 1995). Correlations were interpreted according to the recommendations of Cohen (2013): low (*r* = .10–.29) medium (*r* = .30–.49), or high (*r* ≥ .50). To examine the robustness of correlations, several sensitivity analyses were conducted: (a) Pearson product-moment correlations based on cluster-aggregated teacher ratings, including one aggregated value per cluster to explore effects of non-independent teacher data, (b) Pearson product-moment correlations for parent and child ratings, excluding cases without available teacher ratings to assess the impact of missing teacher reports, and (c) Spearman rank correlations for the whole dataset to account for potential outliers and non-linear associations.

## Results

### Feasibility

Regarding session feasibility, the VR test session lasted for an average of 73.32 min (*SD* = 8.93, *n* = 80). Technical issues occurred in eleven sessions (e.g., low batteries, software crashes, broken cables). On average, resolving these problems took 7.82 min (*SD* = 5.04, *n* = 11) and was rated as having a minor impact on the session (*M* = 0.40, *SD* = 0.52, *n* = 10, scale range = 0–3). All participants underwent all conflict scenarios. For two participants, significant problems occurred (technical problems, incorrect scenario execution by experimenter), leading to a non-evaluation of one scenario each. For two other participants, one scenario was repeated (lack of adherence to scenario instructions). This repeated scenario was included in the further analyses. Participants rated the VR test session as very appealing (*M* = 2.72, *SD* = 0.40, *n* = 81*,* scale range = 0–3). Mean immersion experience scores lay in the midrange of the scale (*M* = 2.28, *SD* = 0.94, *n* = 81, scale range = 0–4), with no significant difference between the clinical and non-clinical sample (*t*(77.13) = 0.01, *p* = .988). Regarding the ecological validity of the VR test scenarios, on average, the children reported that similar situations could likely happen to them or another child in real life (*M* = 1.94–2.51, *SD* = 0.67–0.95, *n* = 80–82, scale range = 0–3) and that they mostly behaved as they would in real life (*M* = 2.21–2.55, *SD* = 0.61–0.83, *n* = 80–82, scale range = 0–3, see supplementary material Table S7). Children’s emotional engagement ranged from low to medium across scenarios (*M* = 0.73–1.76, *SD* = 0.75–0.97, *n* = 79–82, scale range = 0–3). The VR test scenario Defeat in a Competition showed descriptively lower emotional engagement (*M* = 0.73, *SD* = 0.75) compared to the other scenarios (*M* = 1.32–1.76, *SD* = 0.77–0.97). Furthermore, significant differences between the clinical and non-clinical sample were found in ambiguous scenarios, but not hostile scenarios (see supplementary material Table S8-S9). Aggressive behavioral responses were elicited in all VR test scenarios (9.76%–58.03%), with variability depending on the scenario type (ambiguous, hostile) and response (aggressive behavioral response 1, aggressive behavioral response 2). Descriptively, fewer aggressive behavioral responses were evoked in the ambiguous scenarios (9.76%–28.05%) than in the hostile scenarios (26.84%–58.03%). Within scenarios, aggressive behavioral responses tended to be more common in ratings of the second behavioral responses (13.42%–58.03%) than in ratings of the first responses (9.76%–43.91%). Detailed results are provided in the supplementary material (see Table S10). Regarding the exploration of inter-operator effects, Kruskal–Wallis tests revealed no significant differences between the three operators across the aggressive behavioral response scales and SIP scales (clinical sample: *H*(2) = 0.02–2.18, all *p* > .10; non-clinical sample: *H*(2) = 0.12–3.36, all *p* > .10).

### Psychometric Properties

#### Scale Characteristics

Internal consistencies of the aggressive behavioral response scales (α = .85) and SIP scales (α = .86–.95) for the total sample were good to excellent and exceeded the recommended cut-off (α ≥ .70), both in ambiguous scenarios and hostile scenarios. Furthermore, item-total correlations were above the recommended cut-off (*r*_it_ > .30), with one exception (Item: *Hostility*; Scale: *Cue Interpretations*; Scenario: *Insult, r*_it_ = .27). In the clinical sample, the VR test scales showed mostly good to excellent internal consistencies, whereas in the non-clinical sample, estimates were acceptable to good for hostile scenarios but low for several VR test scales (*Aggressive Behavioral Response, Response Access, Response Evaluations*) in ambiguous scenarios (see supplementary material Table S11). Intercorrelations between the VR test scales were medium to high in ambiguous scenarios (*r* = .42–.84) and low to high in hostile scenarios (*r* = .28–.83). Three of the correlations exceeded *r* = .80 (see supplementary material Table S12).

#### Discriminant Validity

In terms of the discriminant validity of the VR test scenarios, the rm-ANOVAs showed significant differences with large effect sizes for scenario type (neutral, ambiguous, hostile) across all VR test scales (F = 73.45–285.34, all *p* < .001, *f* = 0.96–1.94). For all VR test scales, post hoc tests revealed higher scores in hostile scenarios compared to ambiguous scenarios (mean difference = 0.37–0.68, all *p* < .001) and compared to the neutral scenario (mean difference = 0.80–1.69, all *p* < .001). Ambiguous scenarios also yielded higher scores on all VR test scales compared to the neutral scenario (mean difference = 0.15–1.01, all *p* < .001). Detailed results are provided in the supplementary material (see Tables S13–S15).

Regarding the discriminant validity of the VR test scales, Table 1 shows the mean differences between the clinical and non-clinical sample. Scale scores were significantly higher in the clinical sample than in the non-clinical sample (all *p* < .05). Almost all comparisons revealed large effect sizes across both scenario types (*d* = 0.77–1.76), with the exception of the *Cue Interpretations* scale in the hostile scenarios, which showed a medium effect size (*d* = 0.57).

**Table 1:** Comparisons of clinical sample and non-clinical sample on VR test scales

VR Test Scale	Clinical Sample			Non-Clinical Sample			Welch’s t-test		
	*n*	*M*	*SD*	*n*	*M*	*SD*	*t (df)*	*p*	*d*
Ambiguous scenarios									
Aggressive Behavioral Response	41	0.36	0.51	40	0.04	0.09	3.87 (42.57)	< .001	0.85
Cue Interpretations	39	1.46	0.78	39	0.97	0.47	3.38 (62.24)	= .001	0.77
Goals	42	0.91	0.91	40	0.11	0.24	5.51 (46.76)	< .001	1.19
Response Access	42	1.11	0.92	39	0.13	0.16	6.81 (43.70)	< .001	1.46
Response Evaluations	40	1.24	0.61	38	0.48	0.32	7.02 (59.92)	< .001	1.57
Hostile scenarios									
Aggressive Behavioral Response	41	1.32	0.73	40	0.35	0.38	7.48 (60.50)	< .001	1.65
Cue Interpretations	39	2.09	0.64	40	1.76	0.52	2.52 (73.36)	= .014	0.57
Goals	41	1.62	1.08	40	0.53	0.53	5.79 (58.62)	< .001	1.28
Response Access	40	1.48	0.91	40	0.45	0.45	6.39 (57.19)	< .001	1.43
Response Evaluations	39	1.74	0.67	40	0.74	0.45	7.80 (66.22)	< .001	1.76

The aggressive behavioral response scales refer to expert ratings of video-recorded behavior during the VR scenarios; all other scales refer to children's self-reports on social information processing

#### Convergent Validity

Table 2 shows the correlations between the VR test scales and the peer-related aggression scales across informants and scenario type. The aggressive behavioral response scales showed medium to high correlations with peer-related aggression in hostile scenarios (*r* = .31–.64) and low to medium correlations in ambiguous scenarios (*r* = .16–.45). For the SIP scales *Goals*, *Response Access,* and *Response Evaluations*, correlations were medium to high in both ambiguous and hostile scenarios (*r* = .34–.71). The *Cue Interpretations* scale showed overall low to medium correlations, with slightly lower correlations in hostile (*r* = .04–.48) than in ambiguous scenarios (*r* = .17–.46). Additionally, we found a small number of very low correlations regarding the *Disturbance of Social Interaction* scale. Overall, correlations between the VR test scales and the ODD/CD symptom scales across different informants and scenario types revealed a similar pattern of correlations, with slightly lower scale scores (see supplementary material Table S16). Regarding the *CD* scale, we found some outliers, particularly low correlations. Furthermore, sensitivity analyses for teacher ratings and Spearman rank correlations yielded a similar pattern of correlations (see Table 2 and Table S16 for further information).

**Table 2:** Correlations of VR test scales with peer-related aggression scales

VR Test Scale	Parent Ratings					Child Ratings					Teacher Ratings				
	SC	IC	SS	SI	TO	SC	IC	SS	SI	TO	SC	IC	SS	SI	TO
Ambiguous scenarios															
Aggressive Behavioral Response	.34^*^	.29^*^	.34^*^	.45^**^	.36^*^	.41^**^	.28^*^	.30^*^	.22	.35^*^	.28^*^	.34^*^	.24	.16	.29^*^
Cue Interpretations	.27^*^	.34^*^	.27^*^	.22	.29^*^	.46^**^	.41^**^	.40^**^	.20	.43^**^	.31^*^	.43^**^	.37^*^	.17	.36^*^
Goals	.45^**^	.45^**^	.50^**^	.44^**^	49^**^	.62^**^	.59^**^	.62^**^	.41^**^	.64^**^	.47^**^	.58^**^	.46^**^	.34^*^	.51^**^
Response Access	.47^**^	.47^**^	.49^**^	.48^**^	.51^**^	.64^**^	.57^**^	.68^**^	.44^**^	.67^**^	.47^**^	.56^**^	.51^**^	.37^*^	.52^**^
Response Evaluations	.54^**^	.47^**^	.51^**^	.50^**^	.54^**^	.68^**^	.55^**^	.66^**^	.41^**^	.67^**^	.50^**^	.52^**^	.51^**^	.43^**^	.53^**^
Hostile scenarios															
Aggressive Behavioral Response	.56^**^	.53^**^	.56^**^	.61^**^	.59^**^	.57^**^	.56^**^	.64^**^	.31^*^	.61^**^	.50^**^	.49^**^	.46^**^	.32^*^	.49^**^
Cue Interpretations	.20	.29^*^	.24^*^	.07	.23^*^	.32^*^	.31^*^	.35^*^	.04	.31^*^	.35^*^	.48^**^	.37^*^	.26^*^	.39^*^
Goals	.49^**^	.48^**^	.53^**^	.42^**^	.52^**^	.59^**^	.62^**^	.66^**^	.43^**^	.66^**^	.49^**^	.61^**^	.50^**^	.37^*^	.54^**^
Response Access	.49^**^	.45^**^	.54^**^	.58^**^	.54^**^	.62^**^	.56^**^	.71^**^	.47^**^	.67^**^	.47^**^	.50^**^	.50^**^	.43^**^	.51^**^
Response Evaluations	.61^**^	.55^**^	.60^**^	.58^**^	.62^**^	.64^**^	.62^**^	.68^**^	.40^**^	.67^**^	.61^**^	.60^**^	.57^**^	.55^**^	.63^**^

*n* = 77–82 (parent ratings); *n* = 78–82 (child ratings); *n* = 59–64 (teacher ratings). SC = *Disturbance of Social Cognitive Information Processing* scale; IC = *Disturbance of Impulse Control* scale; SS = *Disturbance of Social Skills* scale; SI = *Disturbance of Social Interaction* scale; TO = *Peer-Related Aggression Total* scale. The aggressive behavioral response scales refer to expert ratings of video-recorded behavior during the VR scenarios; all other scales refer to children's self-reports on social information processing

Sensitivity analyses: Correlations with cluster-aggregated teacher ratings showed similar pattern (Δ*r* ≤ .10). Correlations with parent and child ratings excluding cases without available teacher ratings showed similar pattern (Δ*r* ≤ .10); in 3 correlations, .10 < Δ*r* ≤ .20. Spearman rank correlations showed no reversals in direction and mostly small differences (Δ*r* ≤ .10); in 10 correlations, .10 < Δ*r* ≤ .20; in 2 correlations, Δ*r* > .20

Significance was determined using Benjamini–Hochberg-adjusted p-values, with corrections applied separately for ambiguous and hostile scenarios

^*^p < .05, **p < .001

## Discussion

The aim of the present pilot study was to provide an initial evaluation of an interactive VR test for children designed to capture behavioral responses and dysfunctional SIP in ambiguous and hostile peer-related conflict scenarios. Data were collected from 8–12-year-old boys with and without aggressive behavior problems. We began by investigating the feasibility of the VR test, before examining its psychometric properties.

### Feasibility

Technical problems were rare, and when they did occur, they did not significantly impact the sessions. Moreover, almost all scenarios could be successfully carried out with the participants and analyzed. These findings are encouraging given that the implementation of VR is generally considered more complex and error-prone than traditional assessments (e.g., images, audio recordings, videos). The session duration of the VR test (mean 73.32 min) exceeded the length of standard therapy sessions in Germany (usually 50 min). However, longer sessions are not unusual in the context of assessment procedures (e.g., intelligence testing) and the duration did not seem to reduce children’s perceived appeal of the VR test, as they rated it as highly appealing. Similar findings regarding appeal have been reported in studies examining VR-based treatment (Alsem et al., 2021) and assessment (Verhoef et al., 2021) of aggressive behavior problems in children. Considering that children with externalizing problems often lack treatment motivation (Goertz-Dorten et al., 2023), high appeal is a notable strength, as assessment procedures are often the first contact with healthcare services. Immersive experience ratings were slightly above the scale midpoint, aligning with previous research using VR to examine behavior and cognitive processes of children, adolescents, and adults in conflict scenarios (Kim et al., 2020; Verhoef et al., 2022; Westerveld et al., 2025). Creating an immersive experience was essential for our assessment, as the use of VR is considered to overcome the limitations of traditional methods by immersing children in emotionally engaging interactions (Verhoef et al., 2021). Although the standard deviations found in our study indicated individual differences, our results suggest that these differences cannot be attributed to the sample type (clinical vs. non-clinical sample). Ecological validity was supported by children’s reports that the scenarios reflected real-life situations and elicited naturalistic responses. These findings indicate a successful conception of the scenarios and are important in view of the VR test’s purpose of exploring behavior and cognitive processes in conflict scenarios close to children's everyday lives. The mean values of emotional engagement across participants were similar to those reported in previous research with boys in the same age range who underwent ambiguous scenarios (Verhoef et al., 2022), and higher than those found in a sample of adolescents in juvenile detention centers who underwent ambiguous and hostile scenarios (Westerveld et al., 2025). Notably, the first VR test scenario, Defeat in a Competition, showed the lowest emotional engagement. The reasons for this might be related to the scenario content (e.g., least provocative interaction) or the structure of the assessment (e.g., sequence effects). Furthermore, the clinical sample showed higher emotional engagement than the non-clinical sample in ambiguous scenarios but not in hostile scenarios, supporting the validity of the ambiguous scenarios for assessing the hostile intent attribution bias. The differences between the ratings of the first and second behavioral responses indicate that the inclusion of a second provocation could elicit additional aggressive behavioral responses. The differences in aggressive behavioral responses between the ambiguous and hostile scenarios can be explained by the more provocative nature of the hostile scenarios. Our findings regarding aggressive behavioral responses in hostile scenarios (26.84%–58.03%) were similar to the findings of a VR-based assessment in similarly aged children undergoing ambiguous scenarios to measure reactive aggression (38%–58%, Verhoef et al., 2022), whereas our findings regarding aggressive behavioral responses in ambiguous scenarios (9.76%–28.05%) differed from the aforementioned study. One explanation might be that Verhoef et al. (2022) implemented active gaming scenarios and provided a more appropriate operationalization of ambiguous scenarios, which led to higher rates of aggressive behavioral responses. Although the ability to compare these results is constrained by various factors (e.g., unknown length of response observations, differences in the rating procedure and sample characteristics), a refinement of the ambiguous scenarios should be considered in order to elicit more aggressive behavior and reduce substantial floor effects, without compromising the scenarios’ ambiguous nature.

### Psychometric Properties

Overall, our findings on the reliability and validity of the VR test are promising, but nevertheless need to be interpreted with caution. The internal consistencies of the SIP scales in ambiguous scenarios are in line with those reported in previous evaluations of the SIP-AP assessment that used similar items to form SIP scales across eight ambiguous scenarios (Bookhout et al., 2021; Kupersmidt et al., 2011). To date, few studies have collected data on behavioral responses and SIP in hostile scenarios. The present findings revealed good to excellent internal consistencies both for the aggressive behavioral response scales across scenarios and for the SIP scales in hostile scenarios. One possible explanation for the low item-total correlations of the item *Hostility* (Scale: *Cue Interpretations*, Scenario: *Insult*) may be that the children strongly attributed hostile intentions to the virtual avatars but did not simultaneously report such high levels of anger or feelings of being disrespected and rejected. The low internal consistencies for several VR test scales in the ambiguous scenarios within the non-clinical sample may be attributable to items with very low means and limited variance, further supporting the need to refine the ambiguous scenarios. There were some high intercorrelations of the VR test scales, indicating scale overlap, which could be expected to some degree given that the SIP steps are interconnected and build upon one another. Previous research similarly demonstrated high correlations between SIP scales (*r* ≥ .80; Kupersmidt et al., 2011). Our results may be explained by various factors, such as the operationalization of VR test outcomes and content overlap between VR scenarios. Furthermore, calculations regarding single SIP dimensions must be considered exploratory in nature, as factorial validity could not be established. In terms of the discriminant validity of the VR test scenarios, in line with expectation, the neutral scenario produced the lowest levels of aggressive behavioral responses and SIP scores across all VR test scales, followed by ambiguous scenarios and finally hostile scenarios. These results underline the potential of VR to enable the realization of distinct scenario categories (e.g., ambiguous and hostile scenarios), but should also be interpreted with caution, as the order of presentation may have influenced the results. Moreover, between-group comparisons supported the discriminant validity of the VR test scales, as the clinical sample showed higher levels of aggressive behavior and dysfunctional SIP compared to the non-clinical sample, with mostly large effect sizes. The medium effect size found for the *Cue Interpretations* scale in hostile scenarios requires further consideration, and might be due to the fact that even children without aggressive behavior problems were annoyed by the actions of the virtual avatars and attributed hostile intent to them. However, caution is warranted when interpreting discriminant validity analyses of several VR test scales in ambiguous scenarios, due to low internal consistencies in the non-clinical sample. Furthermore, effect sizes may be overestimated given that the sampling strategy did not encompass the full range of symptom severity. Lastly, the correlation analyses between the VR test scales and peer-related aggression as well as the ODD/CD symptom scales indicated convergent validity. Low correlations of the FAVK scales and the *Cue Interpretations* scale in hostile scenarios likely suggest that even non-aggressive children perceived the avatars as hostile. The partly low correlations between the VR test scales and the *Disturbance of Social Interaction* scale may be attributable to the fact that this scale consists of items referring to how the social environment (e.g., parents) reacts to peer-related aggression, and not explicitly to the children’s cognitions, emotions, and behavior. The slightly higher correlations for the peer-related aggression scales compared to the ODD/CD symptom scales are plausible given the higher content overlap between the FAVK scales and the VR test scales. However, calculating correlations with ODD/CD symptom scales represents a more proximal measure of behavior and may be less biased by content overlap than the results of FAVK scales. Finally, the partly low correlations between VR test scales and the *CD* symptom scale may be explained by the heterogeneity of the CD scale, as it also includes items about being cruel to animals or stealing.

### Limitations

Several limitations of this study should be mentioned, which may inform directions for future research. First, the results cannot be generalized across all procedures that aim to assess behavioral responses and SIP in conflict scenarios with children, as procedures differ in terms of behavioral rating approaches, interview questions, and scenario content. Attempts to standardize SIP assessment, for example by Kupersmidt et al. (2011), may represent a valuable approach. In addition, the comparability of research involving VR is limited by the use of different VR environments and technical equipment (Blanco et al., 2024). Second, as our study only included boys, the generalizability of our findings is limited in terms of gender. This approach aligns with numerous studies using hypothetical vignettes to explore aggressive behavior and SIP in male participants (De Castro et al., 2005; Hummer et al., 2023; Kupersmidt et al., 2011; Verhoef et al., 2021, 2022; Westerveld et al., 2025). However, a previous study reported a link between SIP and aggressive behavior in girls and suggested that the factor structure of SIP items is invariant across genders (Bookhout et al., 2021). Moreover, a large-scale evaluation which included female and male participants revealed good psychometric properties of a web-based SIP assessment (Russo‐Ponsaran et al., 2021), and a recent study using VR conflict scenarios involving sibling aggression, which also included both girls and boys, demonstrated the potential of VR to complement self-report methods (van Berkel et al., 2025). Based on these findings, future studies that include participants of different genders would be valuable. Third, in line with previous studies (Verhoef et al., 2022; Westerveld et al., 2025), we employed a fixed order of scenarios. Specifically, we chose to present ambiguous scenarios first, followed by clearly hostile provocation scenarios, assuming that the latter would elicit the strongest emotional responses and should therefore be presented last to minimize potential carry-over effects (Verhoef et al., 2021). However, this approach does not allow order-related biases (e.g., cumulative exposure, learning effects, sensitization, or fatigue) to be controlled for. Accordingly, future studies should consider using counterbalanced or randomized designs to isolate scenario-type effects. Fourth, although all sessions were conducted by trained operators following a standardized script, uncontrolled sources of inter- and intra-operator variability (e.g., in timing, verbal delivery, VR avatar gestures) may have introduced bias. While comparisons across operators provided initial evidence of acceptable inter-operator variability, further standardization should be considered in order to reduce variability and ensure reproducible results across sessions and sites. Fifth, there are some limitations related to the VR test outcomes: Even though the ratings of aggressive behavioral responses showed good to excellent agreement, ratings of social competence might also be important for interventions. Additionally, more objective physiological measures may be useful in order to better understand children’s responses during conflict scenarios and refine diagnostic utility (Westerveld et al., 2025). Regarding the SIP items used, factor analyses were not performed due to the small sample size. While the analyses on internal consistency provide indications of scale reliability, establishing factorial validity is essential for future research. Sixth, our study focused on the assessment of reactive aggression and did not address proactive aggression, despite the apparent potential of VR to improve the assessment of both reactive and proactive SIP patterns in children (Verhoef et al., 2022). We chose this approach because in addition to ambiguous scenarios, we aimed to focus on exploring behavioral responses and SIP in several hostile scenarios, which are also under-researched (Kupersmidt et al., 2011; Li et al., 2013). Seventh, as we focused solely on peer-related conflicts, future research could examine adult-related conflict scenarios (e.g., with teachers or parents), as these also represent challenging situations for children in daily life (Matthys et al., 2001). Indeed, initial research has demonstrated the applicability of adult-related scenarios in VR (Westerveld et al., 2025). Eighth, although our results provide initial positive indications regarding the test’s psychometric properties, several key aspects have not yet been systematically evaluated (e.g., test–retest reliability, predictive validity, calibration metrics for clinical decision-making, associations with structured behavioral observations or ecological momentary assessment) and should be addressed in further research. Future studies may also investigate the generalizability of the VR test across a wider range of subgroups, including those with subthreshold and comorbid presentations. Ninth, our assessment is limited by the lack of adaptation of scenario wording for different cultural groups, as interpretations of social situations may vary across cultural contexts, potentially affecting the generalizability of our findings. Furthermore, future research should systematically examine validity across participants from different racial and ethnic backgrounds, as this was not systematically recorded in the present study. Lastly, IQ in the non-clinical sample was not assessed using a standardized instrument, and future studies should include appropriate procedures to improve assessment accuracy.

### Relevance of Study Findings

The present findings are relevant for both research and clinical practice, as they provide promising evidence that VR-based SIP assessments are feasible and well-accepted, with preliminary support for their psychometric properties, and may encourage further research in this area. The results on emotional engagement and immersion experience indicated benefits of using VR, suggesting that it may be a valuable approach to more closely reflect children’s real-life behavior and cognitions in conflict scenarios. The encouraging findings regarding interrater reliability and psychometric properties of the behavioral observations underline the feasibility of rating specific behavioral responses to conflict scenarios. Compared with other modalities (e.g., pictures, audio recordings, videos), VR offers greater potential to achieve this. Moreover, in addition to ambiguous scenarios, this study provides insights into the feasibility of integrating hostile scenarios into VR-based assessment procedures with children. Along with other studies (Verhoef et al., 2022; Westerveld et al., 2025), our study may serve as a starting point for integrating such VR-based assessments into clinical practice. Although clinical implementation will require the consideration of additional factors that are beyond the scope of the current work (e.g., financial costs, standardization, integration into routine clinical practice, incremental validity over lower-cost assessment methods), our findings highlight the potential of VR as an innovative assessment tool. One major advantage of VR-based procedures is that they include children’s own ratings as a source of information. This is especially valuable given that children can provide unique insights into their own thoughts, feelings, and behaviors in conflict situations, which may not be fully accessible to external observers. In sum, VR could provide appealing, motivational, emotionally engaging assessment procedures that help to capture affect, cognition, and behavior close to everyday life, which can then be addressed in therapy sessions. Furthermore, VR-based assessments may complement and enhance traditional multi-informant approaches. However, further research is needed to examine predictive validity.

## Supplementary Information

Below is the link to the electronic supplementary material.

Supplementary file1 (DOCX 2448 KB)

## Data Availability

Data and codes are available from the last author upon reasonable request.
