# Supplementary material for: Children’s Responses to Peer Conflict Scenarios: Feasibility and Initial Psychometric Properties of an Interactive Virtual Reality Assessment in a Clinical and Non-Clinical Sample
Source: Res Child Adolesc Psychopathol. 2026 Jul 3;54(4):85. doi: 10.1007/s10802-026-01481-8 (PMC13331841; doi:10.1007/s10802-026-01481-8)
Supplement: Supplementary file 1 — Supplementary file1 (DOCX 2448 KB) [file 10802_2026_1481_MOESM1_ESM.docx]

**Children’s Responses to Peer Conflict Scenarios: Feasibility and Initial Psychometric Properties of an Interactive Virtual Reality Assessment in a Clinical and Non-Clinical Sample**

**Research on Child and Adolescent Psychopathology**

Simon Klos^1^, Celina Eva-Maria Müller^1^, Sophie Burwick^1^, Manfred Döpfner^1*^, Anja Görtz-Dorten^1,2*^

^1^ Center for Child and Adolescent Cognitive Behavior Therapy (CEKIP), Faculty of Medicine and University Hospital Cologne, University of Cologne, Cologne, Germany

^2^ Department of Child and Adolescent Psychiatry, Psychosomatics and Psychotherapy, Faculty of Medicine and University Hospital Cologne, University of Cologne, Cologne, Germany

^*^ shared last authors

Corresponding author: Simon Klos, Center for Child and Adolescent Cognitive Behavior Therapy (CEKIP), University Hospital Cologne, Pohligstraße 9, 50969 Cologne, Germany. E-mail: simon.klos@uk-koeln.de


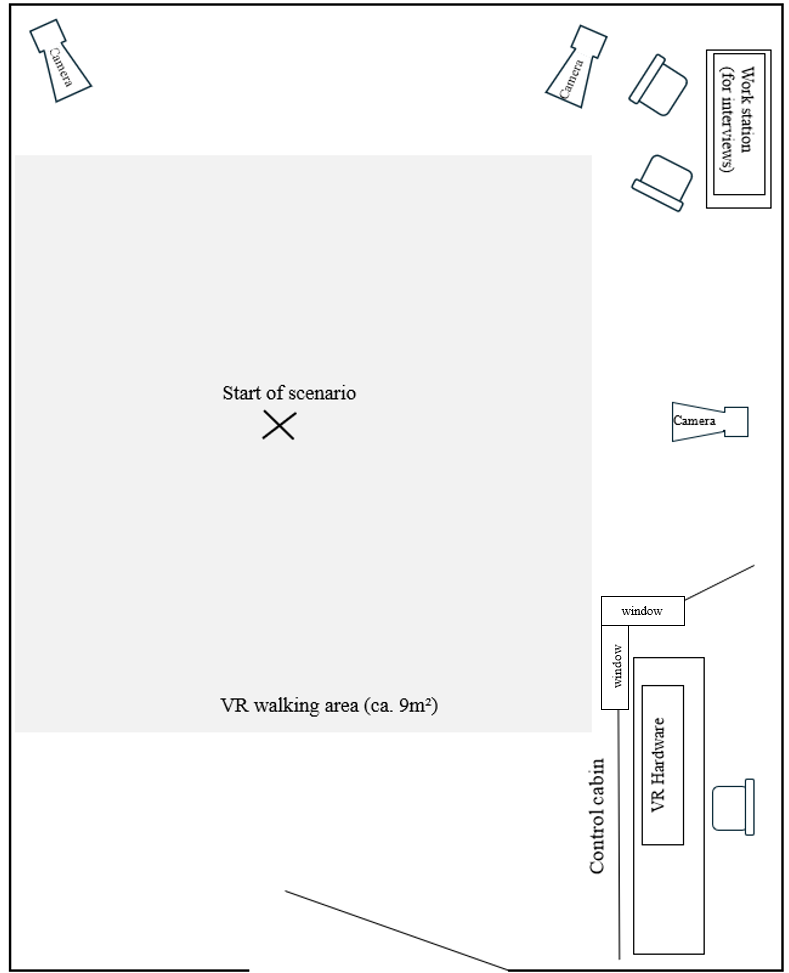


**Figure S1.** Schematic visualization of room specially equipped for VR sessions.

| Category | Scenario | | Location | | Short Description | | | Picture |  |
| --- | --- | --- | --- | --- | --- | --- | --- | --- | --- |
| Neutral Scenario | Small Talk  in a Supermarket | | Supermarket | | The participant visits the supermarket, where an unfamiliar boy asks if he knows where certain products (e.g., candy) are located. The unfamiliar boy responds in a friendly manner to the participant’s responses. | | | 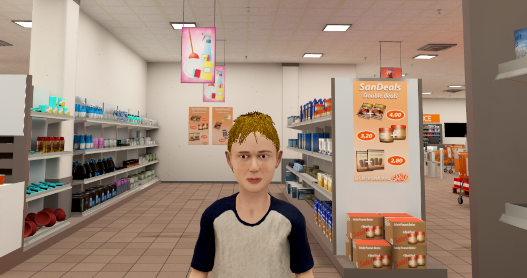 |  |
| Ambiguous Scenarios | Defeat in a Competition | | Park | | The participant plays a game with a familiar boy, Kevin. Both have to take turns naming a color until one of them can't think of any more. Another familiar boy, Tim, stands nearby and watches. Kevin recalls more colors and wins. He celebrates his victory, and Tim praises him. | | | 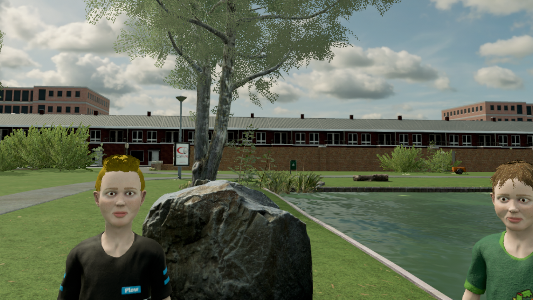 |  |
|  | Untrue Allegation | | Bus stop | | During the VR introductory session, the participant  lent coins for the bus fare to a familiar boy, Patrick. When the participant later asks for the coins back in the VR test session, Patrick claims that he does not remember borrowing any coins. | | | 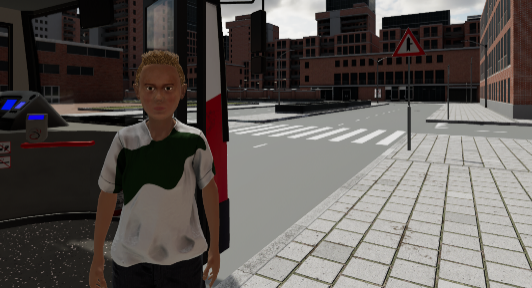 |  |
|  | Social Exclusion | | Playground | | The participant meets two familiar boys, James and Flo, at the playground. The two boys talk about playing on the swings. In the background, one can see that only two swings are available. When the participant asks if he can join in, they answer that this is not possible and that they are doing this without him. | | | ***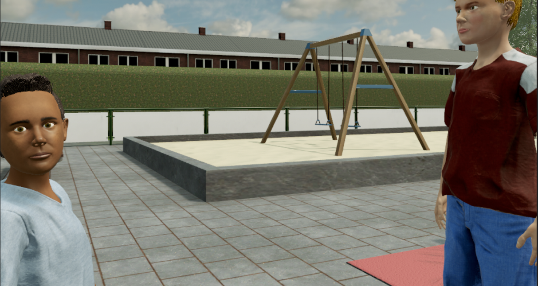*** |  |
| Hostile Scenarios | | Depreciation | | On the bus | | While sitting on the bus, the participant asks two unfamiliar children if they attend the school in Tiviwo. The children actively provoke him and laugh about his clothing and hair. | 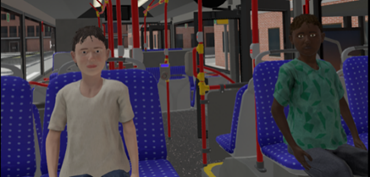 | | |
|  |  | Insult | | Basketball court | | At the basketball court, the participant meets Dominik, a classmate. As he approaches, Dominik accuses the participant of looking at him “like he's stupid” and calls him an “asshole”. | 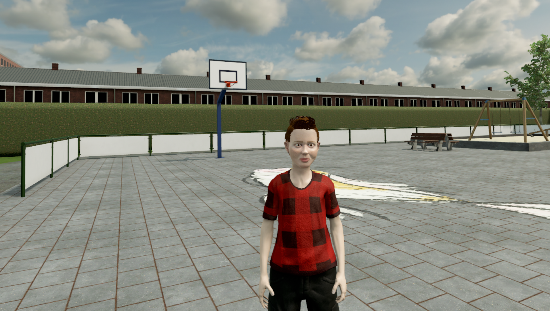 | | |
|  |  | Physical Threat | | Schoolyard | | While waiting alone in the schoolyard for his classmate Till Taff, the participant is approached by an unfamiliar boy who demands coins. The boy actively provokes the participant and threatens him through gestures suggesting physical violence. | 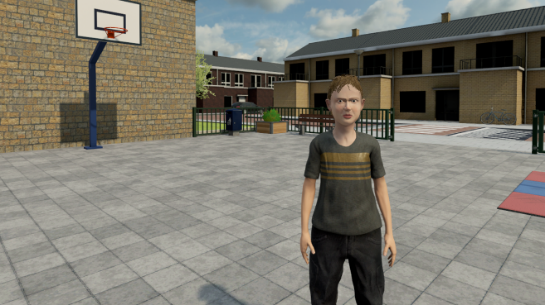 | | |

**Figure S2.** Content descriptions of VR scenarios. *Note.* Images used with permission from CleVR B.V.

| **Name of scenario**  Depreciation | | | 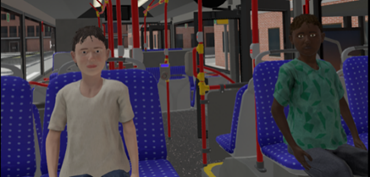 |
| --- | --- | --- | --- |
| **Category**  Hostile scenario | | |  |
| **Location**  On the bus | | |  |
| **Introduction (low-stimulus waiting room)**  *“Imagine this situation happens to you in real life. School has just ended and you are sitting on the school bus on your way home. Two unfamiliar boys are sitting in front of you. They might be from your school. The bus is still at the bus stop and has not yet departed. Please ask them the following question: "Hey, do you go to my school too?”* | | | |
| **Procedure of scenario** | | | |
| (Child asks: *“Hey, do you go to my school too?”*) | | | |
| **Action 1** | 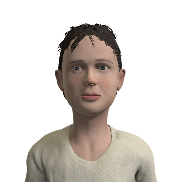 | *“What do you want from us? Your shoes are really ugly.” [laughing at child]* | |
|  | 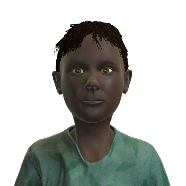 | *"Yeah, ha ha. Did you get them from the clothing drive?" [laughing at child]* | |
| **Aggressive Behavioral Response 1:**  Rating of participant’s behavioral response during and after Action 1 | | | |
| **Action 2** | 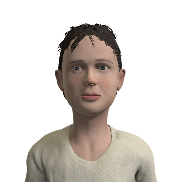 | *“And your hairstyle is totally shit.” [laughing at child]* | |
|  | 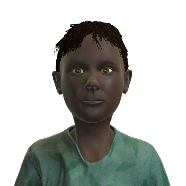 | *"Yeah, ha ha. You should really go to the hairdresser.” [laughing at child]* | |
| **Aggressive Behavioral Response 2:**  Rating of participant’s behavioral response during and after Action 2 | | | |

**Figure S3.** Detailed description of the procedure of an example scenario (depreciation). *Note.* Images used with permission from CleVR B.V.

| Rating scale | Short description | Examples |
| --- | --- | --- |
| 0 = No aggressive behavior | No evidence of verbal or physical aggressive behavior | - “Just leave me alone” - “Do you go to my school or not?” |
| 1 = Minor aggressive behavior | Provocative or impolite behavior initiating or encouraging a conflict | - “Go to the hairdresser yourself, who do you think you are, my dad or something?” - “Old clothes? Take a look at your own. Are they from the '90s?” |
| 2 = Moderate aggressive behavior | Stronger insults (e.g., swear words) or physically provocative behavior | - “Shut the hell up, you little bastards.” |
| 3 = Severe aggressive behavior | Physical threats or acts of physical aggression (e.g., hitting, kicking) | - “Shut up. [Stands up] You want stress?” [Punches] |

**Figure S4.** Examples of responses of the scenario ‘depreciation’ assigned to the category based on the average across all raters.

**Table S1**

*Sample Description*

|  | Clinical sample |  | Non-clinical sample | Comparison ^d^ |
| --- | --- | --- | --- | --- |
| Data collection period | 08/2023–02/2025 |  | 05/2024–02/2025 |  |
| Number of participants (*n*) | 42 |  | 40 |  |
| Mean age (SD) | 10.40 (1.49) |  | 10.51 (1.30) | *t*(79.36)= -0.36, *p* = .723 |
| Education | Primary school: *n* = 20  Secondary school: *n* = 20  Special education school: *n* = 2 |  | Primary school: *n* = 18  Secondary school: *n* = 22 |  |
| ODD/CD diagnosis ^a^ | F90.1: *n* = 22  F91.x: *n* = 19  F92.x: *n* = 01 |  | - |  |
| Mean disruptive symptoms (SD) ^b^ | 0.90 (0.31) |  | 0.12 (0.11) | *t*(51.42) = 15.42, *p* < .001 |
| Mean peer-related aggression (SD) ^c^ | 1.60 (0.39) |  | 0.21 (0.20) | *t*(62.46) = 20.31, *p* < .001 |
| *Note.* ODD = Oppositional defiant disorder; CD = conduct disorder.  ^a^ Reported as ICD-10 codes.  ^b^ Assessed using the parent-rated *Symptom Checklist for Disruptive Behavior Disorders* (SCL-DBD, Doepfner & Goertz-Dorten, 2017).  ^c^ Assessed using the parent-rated *Questionnaire for Aggressive Behavior of Children* (FAVK, Goertz-Dorten & Doepfner, 2021).  ^d^ Conducted using Welch’s t-tests. | | | | |

**Table S2**

*Interrater Reliability of Aggressive Behavioral Responses*

| Scenario type | Absolute Agreement | | Consistency | |
| --- | --- | --- | --- | --- |
|  | Response 1 | Response 2 | Response 1 | Response 2 |
|  | ICC [95% CI] | ICC [95% CI] | ICC [95% CI] | ICC [95% CI] |
| Neutral Scenario | | | | |
| Small Talk in a Supermarket | .75 [.64, .83] | .84 [.77, .89] | .75 [.64, .83] | .84 [.77, .89] |
| Ambiguous Scenarios | | | | |
| Defeat in a Competition | .91 [.88, .94] | .93 [.90, .95] | .92 [.88, .94] | .93 [.90, .95] |
| Untrue Allegation | .74 [.62, .82] | .93 [.90, .96] | .74 [.63, .83] | .93 [.90, .96] |
| Social Exclusion | .93 [.90, .96] | .94 [.92, .96] | .94 [.91, .96] | .95 [.92, .96] |
| Hostile Scenarios |  |  |  |  |
| Depreciation | .93 [.90, .96] | .95 [.92, .97] | .94 [.91, .96] | .95 [.93, .97] |
| Insult | .97 [.95, .98] | .95 [.99, 1.00] | .97 [.96, .98] | .99 [.99, 1.00] |
| Physical Threat | .98 [.97, .99] | .98 [.98, .99] | .98 [.97, .99] | .98 [.98, .99] |
| *Note*. *n* = 81–82. ICC = Intraclass correlation coefficient. All ICCs were conducted using two-way random-effects multiple rater/measurement models. | | | | |

**Table S3**

*Item Characteristics of SIP Items*

| Scale | Description | Item | *n* | *M* | *SD* |
| --- | --- | --- | --- | --- | --- |
| Cue Interpretations | Captures how participants interpreted the scenario, including perceived hostile intent and emotional reactions such as feeling angry, rejected, or disrespected (e.g., “How disrespected did you feel?”) | Hostility | 80 | 1.83 | 0.57 |
|  |  | Rejection | 80 | 1.21 | 0.72 |
|  |  | Disrespect | 79 | 1.58 | 0.70 |
|  |  | Anger | 82 | 1.65 | 0.65 |
| Goals | Captures participants’ goals in the scenario, including motivations to assert dominance, maintain control, or take revenge on others (e.g., “Did you want to get back at [name(s) of avatar(s)/the boys/the boy] or get him/them in trouble?”) | Revenge | 81 | 0.95 | 0.89 |
|  |  | Dominance | 82 | 0.66 | 0.88 |
| Response Access | Captures participants’ access to different behavioral responses in the scenario, including tendencies toward overt aggression, dominance, or relational aggression (e.g., “Would you talk about [name(s) of avatar(s)/the boys/the boy] behind his/their back or try to get other kids not play with him/them?”) | Overt Aggression | 79 | 0.86 | 0.89 |
|  |  | Dominance | 79 | 0.61 | 0.87 |
|  |  | Relational Aggression | 79 | 0.88 | 0.87 |
| Response Evaluations | Captures participants’ evaluation of aggressive responses in the scenario, including confidence in carrying out aggressive behaviors, expected outcomes, and moral judgments (e.g., “How right or wrong would it be to get back at [name(s) of avatar(s)/the boys/the boy]?”) | Antisocial Moral Agency | 79 | 1.00 | 0.76 |
|  |  | Self-Efficacy for Aggression | 79 | 1.49 | 0.94 |
|  |  | Antisocial Outcome Expectancies | 77 | 0.66 | 0.60 |

*Note.* Items were adapted from “*Development and validation of the social information processing application: A Web-based measure of social information processing patterns in elementary school-age boys”,* by J. B. Kupersmidt, R. Stelter, and K. A. Dodge, 2011, Psychological Assessment, *23*(4), p. 838 ([https://doi.org/10.1037/a0023621](https://psycnet.apa.org/doi/10.1037/a0023621)). Several deviations from the original version were implemented. First, we used a 4-point Likert-type scale. Second, items of the first two dimensions were phrased in the past tense, as they were administered immediately after participants experienced each scenario. Items of the last two dimensions were administered after all VR scenarios had been completed. Participants were asked to imagine that the scenario would happen to them and were therefore phrased in hypothetical conditional form. Third, in the *Response Evaluations* dimension, two items (*Self-Efficacy, Antisocial Outcome Expectancy*) were adapted. After the children had undergone all of the six scenarios, they were given four possible responses for each of the six scenarios, from which they could choose which they believed to be the best option: socially competent, socially insecure, verbally aggressive, or physically aggressive. The *Self-Efficacy* and *Antisocial Outcome Expectancy* items referred to the physically aggressive response only if it was selected as the best response. If the verbally aggressive alternative was selected as the best response, or if the socially competent or socially insecure options were chosen, these items referred to the verbally aggressive alternative. Analyses of participants’ choices of the best response (e.g., socially competent, socially insecure) are not included in this paper.

**Table S4**

*Scale Characteristics of Parent Rating Scales*

| Construct |  | Total sample | | | | | Clinical Sample | | | | Non-clinical Sample | | | |
| --- | --- | --- | --- | --- | --- | --- | --- | --- | --- | --- | --- | --- | --- | --- |
|  |  | *n* | *M* | *SD* | ɑ [95% CI] |  | *n* | *M* | *SD* |  | *n* | *M* | *SD* |  |
| Peer-related aggression ^a^ | SC | 81 | 1.07 | 0.86 | .94 [.91, .95] |  | 41 | 1.82 | 0.50 |  | 40 | 0.31 | 0.30 |  |
|  | IC | 82 | 1.16 | 0.99 | .92 [.88, .94] |  | 42 | 2.02 | 0.54 |  | 40 | 0.26 | 0.33 |  |
|  | SS | 82 | 0.98 | 0.92 | .95 [.93, .96] |  | 42 | 1.75 | 0.61 |  | 40 | 0.18 | 0.26 |  |
|  | SI | 81 | 0.44 | 0.52 | .85 [.79, .90] |  | 41 | 0.81 | 0.49 |  | 40 | 0.07 | 0.14 |  |
|  | TO | 82 | 0.92 | 0.77 | .97 [.96, .98] |  | 42 | 1.60 | 0.39 |  | 40 | 0.21 | 0.20 |  |
| ODD/CD symptoms ^b^ | ODD | 82 | 1.06 | 0.88 | .94 [.92, .96] |  | 42 | 1.83 | 0.53 |  | 40 | 0.25 | 0.20 |  |
|  | IRR | 82 | 1.30 | 1.04 | .92 [.88, .95] |  | 42 | 2.17 | 0.64 |  | 40 | 0.39 | 0.37 |  |
|  | HS | 82 | 0.92 | 0.85 | .91 [.87, .94] |  | 42 | 1.63 | 0.60 |  | 40 | 0.18 | 0.20 |  |
|  | CD | 82 | 0.38 | 0.48 | .76 [.67, .83] |  | 42 | 0.70 | 0.49 |  | 40 | 0.05 | 0.10 |  |
| *Note*. SC = *Disturbance of Social Cognitive Information Processing* scale; IC = *Disturbance of Impulse Control* scale; SS = *Disturbance of Social Skills* scale; SI = *Disturbance of Social Interaction* scale; TO = *Peer-Related Aggression Total* scale; ODD = *ODD Total* scale; IRR = *ODD Irritable* scale; HS = *ODD Headstrong* scale; CD = *CD* scale.  ^a^ Assessed using the parent-rated *Questionnaire for Aggressive Behavior of Children* (FAVK, Goertz-Dorten & Doepfner, 2021).  ^b^ Assessed using the parent-rated *Symptom Checklist for Disruptive Behavior Disorders* (SCL-DBD, Doepfner & Goertz-Dorten, 2017). | | | | | | | | | | | | | | |

**Table S5**

*Scale Characteristics of Child Rating Scales*

| Construct |  | Total sample | | | | | Clinical Sample | | | | Non-clinical Sample | | | |
| --- | --- | --- | --- | --- | --- | --- | --- | --- | --- | --- | --- | --- | --- | --- |
|  |  | *n* | *M* | *SD* | ɑ [95% CI] |  | *n* | *M* | *SD* |  | *n* | *M* | *SD* |  |
| Peer-related aggression ^a^ | SC | 82 | 0.81 | 0.62 | .84 [.78, .88] |  | 42 | 1.24 | 0.55 |  | 40 | 0.40 | 0.26 |  |
|  | IC | 82 | 0.97 | 0.82 | .83 [.76, .88] |  | 42 | 1.55 | 0.68 |  | 40 | 0.36 | 0.38 |  |
|  | SS | 82 | 0.74 | 0.73 | .89 [.85, .93] |  | 42 | 1.24 | 0.66 |  | 40 | 0.21 | 0.28 |  |
|  | SI | 82 | 0.60 | 0.50 | .64 [.51, .75] |  | 42 | 0.76 | 0.55 |  | 40 | 0.43 | 0.40 |  |
|  | TO | 82 | 0.77 | 0.58 | .94 [.92, .96] |  | 42 | 1.19 | 0.51 |  | 40 | 0.34 | 0.23 |  |
| ODD/CD symptoms ^b^ | ODD | 81 | 0.87 | 0.67 | .87 [.83, .91] |  | 41 | 1.35 | 0.55 |  | 40 | 0.38 | 0.35 |  |
|  | IRR | 81 | 1.16 | 0.90 | .86 [.80, .91] |  | 41 | 1.76 | 0.77 |  | 40 | 0.55 | 0.53 |  |
|  | HS | 81 | 0.70 | 0.63 | .78 [.69, .85] |  | 41 | 1.11 | 0.58 |  | 40 | 0.28 | 0.31 |  |
|  | CD | 80 | 0.35 | 0.44 | .74 [.63, .82] |  | 40 | 0.60 | 0.49 |  | 40 | 0.11 | 0.20 |  |
| *Note*. SC = *Disturbance of Social Cognitive Information Processing* scale; IC = *Disturbance of Impulse Control* scale; SS = *Disturbance of Social Skills* scale; SI = *Disturbance of Social Interaction* scale; TO = *Peer-Related Aggression Total* scale; ODD = *ODD Total* scale; IRR = *ODD Irritable* scale; HS = *ODD Headstrong* scale; CD = *CD* scale.  ^a^ Assessed using the parent-rated *Questionnaire for Aggressive Behavior of Children* (FAVK, Goertz-Dorten & Doepfner, 2021).  ^b^ Assessed using the parent-rated *Symptom Checklist for Disruptive Behavior Disorders* (SCL-DBD, Doepfner & Goertz-Dorten, 2017). | | | | | | | | | | | | | | |

**Table S6**

*Scale Characteristics of Teacher Rating Scales*

| Construct |  | Total sample | | | | | Clinical Sample | | | | Non-clinical Sample | | | |
| --- | --- | --- | --- | --- | --- | --- | --- | --- | --- | --- | --- | --- | --- | --- |
|  |  | *n* | *M* | *SD* | ɑ [95% CI] |  | *n* | *M* | *SD* |  | *n* | *M* | *SD* |  |
| Peer-related aggression ^a^ | SC | 63 | 1.09 | 0.91 | .94 [.92, .96] |  | 35 | 1.74 | 0.62 |  | 28 | 0.28 | 0.43 |  |
|  | IC | 64 | 0.90 | 0.92 | .89 [.84, .93] |  | 36 | 1.56 | 0.74 |  | 28 | 0.14 | 0.31 |  |
|  | SS | 64 | 0.90 | 0.93 | .94 [.92, .96] |  | 36 | 1.55 | 0.72 |  | 28 | 0.16 | 0.46 |  |
|  | SI | 64 | 0.50 | 0.59 | .84 [.77, .89] |  | 36 | 0.83 | 0.57 |  | 28 | 0.14 | 0.34 |  |
|  | TO | 64 | 0.86 | 0.79 | .97 [.96, .98] |  | 36 | 1.44 | 0.55 |  | 28 | 0.19 | 0.37 |  |
| ODD/CD symptoms ^b^ | ODD | 64 | 1.10 | 0.97 | .95 [.93, .97] |  | 36 | 1.78 | 0.66 |  | 28 | 0.24 | 0.46 |  |
|  | IRR | 64 | 1.26 | 1.12 | .93 [.90, .96] |  | 36 | 2.04 | 0.82 |  | 28 | 0.25 | 0.45 |  |
|  | HS | 64 | 1.02 | 0.94 | .93 [.90, .95] |  | 36 | 1.63 | 0.72 |  | 28 | 0.23 | 0.49 |  |
|  | CD | 64 | 0.56 | 0.70 | .84 [.76, .89] |  | 36 | 0.90 | 0.68 |  | 28 | 0.12 | 0.43 |  |
| *Note*. SC = *Disturbance of Social Cognitive Information Processing* scale; IC = *Disturbance of Impulse Control* scale; SS = *Disturbance of Social Skills* scale; SI = *Disturbance of Social Interaction* scale; TO = *Peer-Related Aggression Total* scale; ODD = *ODD Total* scale; IRR = *ODD Irritable* scale; HS = *ODD Headstrong* scale; CD = *CD* scale.  ^a^ Assessed using the parent-rated *Questionnaire for Aggressive Behavior of Children* (FAVK, Goertz-Dorten & Doepfner, 2021).  ^b^ Assessed using the parent-rated *Symptom Checklist for Disruptive Behavior Disorders* (SCL-DBD, Doepfner & Goertz-Dorten, 2017). | | | | | | | | | | | | | | |

**Table S7**

*Ecological Validity – Item Characteristics*

| Item | Ambiguous scenarios | | | | | | | |  | | Hostile scenarios | | | | | | | | |
| --- | --- | --- | --- | --- | --- | --- | --- | --- | --- | --- | --- | --- | --- | --- | --- | --- | --- | --- | --- |
|  | Defeat in a Competition | | | Untrue Allegation | | | Social Exclusion | |  | | Depreciation | | | Insult | | Physical Threat | | | |
|  | *M* | *SD* | *M* | | *SD* | *M* | | *SD* | |  | | *M* | *SD* | *M* | *SD* | | *M* | *SD* | |
| “Do you think something like that could happen to you or another child in real life?” | 2.20 | 0.88 | 2.12 | | 0.95 | 2.51 | | 0.67 | |  | | 2.17 | 0.76 | 2.14 | 0.79 | | 1.94 | 0.86 | |
| “Did you behave in this scenario the same way you would with other children your age?” | 2.34 | 0.69 | 2.21 | | 0.83 | 2.37 | | 0.68 | |  | | 2.44 | 0.67 | 2.38 | 0.66 | | 2.55 | 0.61 | |
| *Note. n* = 80–82. Scale range of items 0–3. | | | | | | | | | | | | | | | | | | |  |

**Table S8**

*Emotional Engagement – Item Characteristics*

| Item | Ambiguous scenarios | | | | | |  | Hostile scenarios | | | | | |
| --- | --- | --- | --- | --- | --- | --- | --- | --- | --- | --- | --- | --- | --- |
|  | Defeat in a Competition | | Untrue  Allegation | | Social  Exclusion | |  | Depreciation | | Insult | | Physical Threat | |
|  | *M* | *SD* | *M* | *SD* | *M* | *SD* |  | *M* | *SD* | *M* | *SD* | *M* | *SD* |
| “How angry did you feel when this happened to you?” | 0.98 | 0.86 | 1.80 | 0.79 | 1.61 | 0.87 |  | 1.90 | 0.94 | 1.98 | 1.04 | 1.63 | 1.11 |
| “How much did you care when this happened to you?” | 0.54 | 0.84 | 0.98 | 0.98 | 1.14 | 0.90 |  | 1.42 | 1.02 | 1.55 | 1.05 | 0.99 | 1.14 |
| Emotional Engagement | 0.73 | 0.75 | 1.38 | 0.77 | 1.36 | 0.78 |  | 1.66 | 0.84 | 1.76 | 0.94 | 1.32 | 0.97 |
| *Note. n* = 79–82. Scale range of items 0–3. | | | | | | | | | | | | | |

**Table S9**

*Comparisons of Clinical Sample and Non-Clinical Sample on Emotional Engagement*

| VR Test Scenario | Clinical Sample | | |  | Non-Clinical Sample | | |  | Welch’s t-test | | |
| --- | --- | --- | --- | --- | --- | --- | --- | --- | --- | --- | --- |
|  | *n* | *M* | *SD* |  | *n* | *M* | *SD* |  | *t (df)* | *p* | *d* |
| Ambiguous scenarios | | | | | | | | | | | |
| Defeat in a Competition | 40 | 0.95 | 0.87 |  | 40 | 0.50 | 0.53 |  | 2.86 (64.41) | .006 | 0.64 |
| Untrue Allegation | 40 | 1.68 | 0.87 |  | 40 | 1.07 | 0.53 |  | 3.75 (64.28) | <.001 | 0.84 |
| Social Exclusion | 40 | 1.55 | 0.89 |  | 40 | 1.16 | 0.60 |  | 2.28 (68.59) | .026 | 0.51 |
| Hostile scenarios | | | | | | | | | | | |
| Depreciation | 39 | 1.79 | 0.91 |  | 40 | 1.54 | 0.75 |  | 1.37 (73.78) | .175 | 0.31 |
| Insult | 40 | 1.93 | 0.98 |  | 40 | 1.60 | 0.88 |  | 1.56 (77.14) | .122 | 0.35 |
| Physical Threat | 39 | 1.45 | 1.09 |  | 40 | 1.20 | 0.83 |  | 1.14 (70.93) | .259 | 0.26 |

**Table S10**

*Aggressive Behavioral Responses – Absolute and Relative Frequencies*

| Item |  |  |  | | Ambiguous scenarios | | |  | Hostile scenarios | | | | | | |
| --- | --- | --- | --- | --- | --- | --- | --- | --- | --- | --- | --- | --- | --- | --- | --- |
|  |  | Defeat in a Competition | | | Untrue  Allegation | | Social  Exclusion | | Depreciation | | Insult | | Physical Threat | | |
|  |  | *n* | | % | *n* | % | *n* | % | *n* | % | *n* | % | *n* | % | |
| Aggressive behavioral response 1 | None | 70 | | 86.42 | 73 | 89.02 | 74 | 90.24 | 46 | 56.10 | 60 | 73.17 | 47 | 57.32 | |
|  | Minor | 9 | | 11.11 | 9 | 10.98 | 6 | 7.32 | 29 | 35.37 | 9 | 10.98 | 16 | 19.51 | |
|  | Moderate | 1 | | 1.23 | - | - | 1 | 1.22 | 7 | 8.54 | 8 | 9.76 | 10 | 12.20 | |
|  | Severe | 1 | | 1.23 | - | - | 1 | 1.22 | - | - | 5 | 6.10 | 9 | 10.98 | |
|  |  |  | |  |  |  |  |  |  |  |  |  |  |  | |
| Aggressive behavioral response 2 | None | 70 | | 86.42 | 71 | 86.59 | 59 | 71.95 | 37 | 45.12 | 34 | 41.98 | 36 | 43.90 | |
|  | Minor | 7 | | 8.64 | 7 | 8.54 | 17 | 20.73 | 32 | 39.02 | 16 | 19.75 | 9 | 10.98 | |
|  | Moderate | 3 | | 3.70 | 3 | 3.66 | 2 | 2.44 | 10 | 12.20 | 10 | 12.35 | 9 | 10.98 | |
|  | Severe | 1 | | 1.23 | 1 | 1.22 | 4 | 4.88 | 3 | 3.66 | 21 | 25.93 | 28 | 34.15 | |
| Note. Frequencies are average ratings across all three raters categorized as follows: < 0.50 = *No aggressive behavior*; ≥ 0.50 and < 1.50 = *Minor aggressive behavior*; ≥ 1.50 and < 2.50 = *Moderate aggressive behavior*; ≥ 2.50 = *Severe aggressive behavior.* | | | | | | | | | | | | | | |  |

**Table S11**

*Internal Consistencies of VR Test Scales*

|  |  | | Total Sample | | | Clinical Sample | | Non-clinical Sample | |
| --- | --- | --- | --- | --- | --- | --- | --- | --- | --- |
|  | No. of items | | ɑ [95% CI] | Ω [95% CI] | *r*_it (range)_ | ɑ [95% CI] | Ω [95% CI] | ɑ [95% CI] | Ω [95% CI] |
| Ambiguous scenarios |  |  |  |  |  |  |  |  |  |
| Aggressive Behavioral Response | 6 |  | .85 [.80, .90] | .87 [.76, .93] | .55–.73 | .85 [.76, .91] | .86 [.71, .93] | .48 [.18, .69] | - ^a^ |
| Cue Interpretations | 12 |  | .92 [.89, .95] | .92 [.87, .95] | .62–.73 | .93 [.89, .96] | .92 [.86, .95] | .87 [.80, .92] | .86 [.75, .91] |
| Goals | 6 |  | .91 [.88, .94] | .92 [.87, 95] | .60–.84 | .89 [.83, .94] | .89 [.81, .94] | .80 [.69, .88] | - ^a^ |
| Response Access | 9 |  | .95 [.93, .97] | .95 [.91, .97] | .76–.88 | .93 [.90, .96] | .93 [.88, .96] | .64 [.44, .79] | - ^a^ |
| Response Evaluations | 9 |  | .86 [.81, .90] | .88 [.82, .91] | .30–.72 | .82 [.72, .89] | .82 [.72, .95] | .67 [.48, .81] | - ^a^ |
| Hostile scenarios |  |  |  |  |  |  |  |  |  |
| Aggressive Behavioral Response | 6 |  | .85 [.79, .90] | .87 [.81, .90] | .56–.72 | .79 [.66, .87] | .79 [.65, .87] | .69 [.52, .82] | .71 [.52, .87] |
| Cue Interpretations | 12 |  | .86 [.80, .90] | .85 [.76, .90] | .27–.69 | .86 [.78, .92] | .86 [.78, .94] | .83 [.73, .90] | .80 [.65, .93] |
| Goals | 6 |  | .94 [.91, .96] | .94 [.91, .96] | .77–.87 | .93 [.90, .96] | .93 [.89, .96] | .85 [.77, .91] | .85 [.70, .92] |
| Response Access | 9 |  | .95 [.93, .96] | .94 [.92, .96] | .70–.88 | .93 [.90, .96] | .93 [.88, .96] | .88 [.82, .93] | .89 [.75, .94] |
| Response Evaluations | 9 |  | .90 [.86, .93] | .89 [.85, .92] | .50–.77 | .84 [.76, .91] | .83 [.71, .90] | .78 [.66, .87] | .74 [.54, .86] |
| *Note. n* = 78–82 (total sample), *n* = 39–42 (clinical sample), *n* = 38–40 (non-clinical sample). ^a^ Estimates could not be computed since division-by-zero errors were encountered in the matrix procedure. | | | | | | | | | |

**Table S12**

*Intercorrelations of VR Test Scales*

|  | Aggressive  Behavioral Response | Cue Interpretations | Goals | Response Access | Response Evaluations |
| --- | --- | --- | --- | --- | --- |
| Aggressive Behavioral Response | – | .37^**^ | .66^**^ | .75^**^ | .74^**^ |
| Cue Interpretations | .42^**^ | – | .55^**^ | .36^*^ | .28^**^ |
| Goals | .57^**^ | .67^**^ | – | .79^**^ | .71^**^ |
| Response Access | .58^**^ | .70^**^ | .84^**^ | – | .83^**^ |
| Response Evaluations | .53^**^ | .64^**^ | .71^**^ | .83^**^ | – |
| *Note.* *n* = 74–81. Scale correlations for ambiguous scenarios below the diagonal; scale correlations for hostile scenarios above the diagonal. The aggressive behavioral response scales refer to expert ratings of video-recorded behavior during the VR scenarios; all other scales refer to children's self-reports on social information processing.  ** p* < .05*, **p* < .001*.* | | | | | |

**Table S13**

*Item Characteristics of VR Test scales for Neutral, Ambiguous, and Hostile Scenarios*

| VR Test Scale | Neutral scenario | | |  | Ambiguous scenarios | | |  | Hostile scenarios | | |  |
| --- | --- | --- | --- | --- | --- | --- | --- | --- | --- | --- | --- | --- |
|  | *n* | *M* | *SD* |  | *n* | *M* | *SD* |  | *n* | *M* | *SD* |  |
| Aggressive Behavioral Response | 82 | 0.04 | 0.21 |  | 81 | 0.20 | 0.40 |  | 81 | 0.84 | 0.76 |  |
| Cue Interpretations | 80 | 0.21 | 0.45 |  | 78 | 1.22 | 0.68 |  | 79 | 1.92 | 0.60 |  |
| Goals | 82 | 0.11 | 0.41 |  | 82 | 0.52 | 0.78 |  | 81 | 1.08 | 1.01 |  |
| Response Access | 82 | 0.08 | 0.35 |  | 81 | 0.64 | 0.83 |  | 80 | 0.96 | 0.88 |  |
| Response Evaluations | 82 | 0.40 | 0.42 |  | 78 | 0.87 | 0.62 |  | 79 | 1.23 | 0.76 |  |
| *Note.* The aggressive behavioral response scales refer to expert ratings of video-recorded behavior during the VR scenarios; all other scales refer to children's self-reports on social information processing. | | | | | | | | | | | |  |

**Table S14**

*Results rm-ANOVAs – Comparison of Neutral, Ambiguous, and Hostile Scenarios on VR Test Scales*

| VR Test Scale |  | rm-ANOVA | | |
| --- | --- | --- | --- | --- |
|  |  | *F*(*df1, df2*) | *p* | *f* |
| Aggressive Behavioral Response |  | 088.52 (1.27, 100.09)^a^ | <.001 | 1.06 |
| Cue Interpretations |  | 285.34 (2.00, 146.00) | <.001 | 1.94 |
| Goals |  | 073.69 (1.41, 113.09)^a^ | <.001 | 0.96 |
| Response Access |  | 073.45 (1.42, 110.82)^a^ | <.001 | 0.98 |
| Response Evaluations |  | 097.24 (1.67, 125.52)^a^ | <.001 | 1.15 |
| *Note. n* = 74–81*.* The aggressive behavioral response scales refer to expert ratings of video-recorded behavior during the VR scenarios; all other scales refer to children's self-reports on social information processing.  ^a^ Due to violations of sphericity (Mauchly’s test *p* < .05), Greenhouse-Geisser correction was applied. | | | | |

**Table S15**

*Results of Post Hoc Tests – Comparison of Neutral, Ambiguous, and Hostile Scenarios on VR Test Scales*

| VR Test Scale | Group 1 | Group 2 |  | Mean Difference [95% CI] |  |  |
| --- | --- | --- | --- | --- | --- | --- |
| Aggressive Behavioral Response | AS | NS |  | 0.15 [0.07, 0.24] |  |  |
|  | HS | NS |  | 0.80 [0.60, 0.99] |  |  |
|  | HS | AS |  | 0.64 [0.48, 0.81] |  |  |
| Cue Interpretations | AS | NS |  | 1.01 [0.85, 1.18] |  |  |
|  | HS | NS |  | 1.69 [1.51, 1.87] |  |  |
|  | HS | AS |  | 0.68 [0.50, 0.86] |  |  |
| Goals | AS | NS |  | 0.41 [0.24, 0.59] |  |  |
|  | HS | NS |  | 0.99 [0.73, 1.24] |  |  |
|  | HS | AS |  | 0.57 [0.42, 0.73] |  |  |
| Response Access | AS | NS |  | 0.56 [0.36, 0.76] |  |  |
|  | HS | NS |  | 0.93 [0.70, 1.16] |  |  |
|  | HS | AS |  | 0.37 [0.25, 0.49] |  |  |
| Response Evaluations | AS | NS |  | 0.48 [0.33, 0.62] |  |  |
|  | HS | NS |  | 0.86 [0.68, 1.04] |  |  |
|  | HS | AS |  | 0.39 [0.26, 0.51] |  |  |
| *Note. n* = 74–81*.* NS = Neutral scenario; AS = Ambiguous scenarios; HS = Hostile scenarios. The aggressive behavioral response scales refer to expert ratings of video-recorded behavior during the VR scenarios; all other scales refer to children's self-reports on social information processing. All *p* < .001. | | | | |  |  |

**Table S16**

*Correlations of VR Test Scales with ODD/CD Symptom Scales*

| VR Test Scale | Parent Ratings | | | | |  |  | Child Ratings | | | |  | Teacher Ratings | | | |
| --- | --- | --- | --- | --- | --- | --- | --- | --- | --- | --- | --- | --- | --- | --- | --- | --- |
|  | ODD | | IRR | HS | CD |  |  | ODD | IRR | HS | CD |  | ODD | IRR | HS | CD |
| Ambiguous scenarios | |  |  |  |  |  |  |  |  |  |  |  |  |  |  |  |
| Aggressive Behavioral Response | .36^**^ | | .30^**^ | .38^*^ | .32^*^ |  |  | .29^**^ | .20^**^ | .33^**^ | .28^**^ |  | .24^**^ | .25^*^ | .22^**^ | .14^**^ |
| Cue Interpretations | .30^**^ | | .31^**^ | .27^**^ | .13^**^ |  |  | .33^**^ | .32^**^ | .29^**^ | .21^**^ |  | .26^*^ | .40^*^ | .14^**^ | .02^**^ |
| Goals | .49^**^ | | .48^**^ | .46^**^ | .34^**^ |  |  | .59^**^ | .53^**^ | .56^**^ | .48^**^ |  | .42^*^ | .51^**^ | .32^**^ | .24^**^ |
| Response Access | .51^**^ | | .48^**^ | .50^**^ | .38^*^ |  |  | .55^**^ | .51^**^ | .50^**^ | .51^**^ |  | .44^**^ | .50^**^ | .37^**^ | .31^**^ |
| Response Evaluations | .54^**^ | | .51^**^ | .53^**^ | .44^**^ |  |  | .54^**^ | .48^**^ | .52^**^ | .48^**^ |  | .49^**^ | .52^**^ | .43^*^ | .36^*^ |
| Hostile scenarios | | | | | | | | | | | | | | | | |
| Aggressive Behavioral Response | .61^**^ | | .54^**^ | .61^**^ | .51^**^ |  |  | .51^**^ | .42^**^ | .52^**^ | .49^**^ |  | .47^**^ | .50^**^ | .42^**^ | .35^*^ |
| Cue Interpretations | .21^**^ | | .20^**^ | .21^**^ | .18^**^ |  |  | .23^**^ | .25^**^ | .19^**^ | .12^**^ |  | .29^**^ | .34^**^ | .23^**^ | .23^**^ |
| Goals | .50^**^ | | .47^**^ | .50^**^ | .37^**^ |  |  | .59^**^ | .54^**^ | .57^**^ | .50^**^ |  | .51^**^ | .59^**^ | .41^**^ | .28^**^ |
| Response Access | .50^**^ | | .43^**^ | .51^**^ | .44^**^ |  |  | .56^**^ | .50^**^ | .53^**^ | .53^**^ |  | .49^**^ | .50^**^ | .46^**^ | .43^**^ |
| Response Evaluations | .59^**^ | | .56^**^ | .58^**^ | .51^**^ |  |  | .63^**^ | .59^**^ | .58^**^ | .58^**^ |  | .61^**^ | .60^**^ | .58^**^ | .49^**^ |
| *Note. n* = 78–82 (parent ratings), *n* = 76–81 (child ratings), *n* = 60–64 (teacher ratings). ODD = *ODD Total* scale; IRR = *ODD Irritable* scale; HS = *ODD Headstrong* scale; CD = *CD* scale. The aggressive behavioral response scales refer to expert ratings of video-recorded behavior during the VR scenarios; all other scales refer to children's self-reports on social information processing.  Sensitivity analyses: Correlations with cluster-aggregated teacher ratings showed a similar pattern (Δ*r* ≤ .10). Correlations with parent and child ratings excluding cases without available teacher ratings showed similar pattern (Δ*r* ≤ .10). Spearman rank correlations showed no reversals in direction and mostly small differences (*r* ≤ .10); in 15 correlations, .10 < Δ*r* ≤ .20; in 2 correlations, Δ*r* > .20.  Significance was determined using Benjamini–Hochberg-adjusted p-values, with corrections applied separately for ambiguous and hostile scenarios.  ** p* < .05*, **p* < .001*.* | | | | | | | | | | | | | | | | |
